# Do student differences in reading enjoyment relate to achievement when using the random-intercept cross-lagged panel model across primary and secondary school?

**DOI:** 10.1371/journal.pone.0285739

**Published:** 2023-06-09

**Authors:** William Luya Coventry, Sarah Farraway, Sally A. Larsen, Tim P. Enis, Alexander Q. Forbes, Stephen L. Brown

**Affiliations:** 1 School of Psychology, University of New England, Armidale, NSW, Australia; 2 School of Education, University of New England, Armidale, NSW, Australia; Chulalongkorn University, THAILAND

## Abstract

Recent longitudinal research using the random-intercept cross-lagged panel model (RI-CLPM), which disentangles the within and between variances, has afforded greater insights than previously possible. Moreover, the impact of reading enjoyment and reading for fun on subsequent school achievement, and vice versa, has only recently been scrutinized through this lens. This study’s longitudinal data (grades 3, 5, 7, and 9) comprised 2,716 Australian students aged 8 to 16 years, with school reading achievement measured by the National Assessment Program: Literacy and Numeracy (NAPLAN). The RI-CLPMs’ within-person effects were not trivial, accounting for approximately two-thirds and one-third of the variance in enjoyment/fun and achievement, respectively, with between-person effects accounting for the balance. Here, we highlight a reversing direction of reading achievement’s cross-lagged effect on subsequent reading enjoyment but note that the evidence for this over a reciprocal directionality was marginal. In mid-primary school, achievement at grade 3 predicted enjoyment at grade 5 more than the converse (i.e. enjoyment at grade 3 to achievement at grade 5). By secondary school, however, the directionality had flipped: enjoyment at grade 7 predicted achievement at grade 9 more so than the reverse. We termed this pattern the skill-leisure-skill directionality (S-L-S), as it concurred with the only two former studies that modelled equivalent instruments with the RI-CLPM. This model’s cross-lagged estimates represent deviations relative to a student’s average (i.e., within-person effect). In other words, students who enjoyed reading more (or less) in grade 7 achieved reading scores that were higher (or lower) than their average in grade 9. The implications for reading pedagogy are further discussed.

## Introduction

Children differ in their enjoyment of reading. For example, for each word read by those disinclined to read, ardent readers read upwards of 200 words [1]. We assess how such differences in reading enjoyment and reading for fun relate to school achievement. Perhaps higher reading achievement gives rise to students enjoying or having more fun with their reading (*skill to enjoy*). Inversely, enjoyment/fun may lead to higher achievement (*enjoy to skill*). Indeed, the effects may be reciprocal [2]. Pragmatically, this can depend on the child’s age; for instance, the *skill to enjoy* directionality can reverse when the student reaches a more mature stage of reading. We evaluate the literature, focusing on longitudinal studies that are well-placed to address directional changes with age. In so doing, since no other longitudinal studies of reading enjoyment or reading for fun exist, we consider the broader literature to which these items belong, which we term ‘leisure’ reading. This broader literature has regularly encompassed reading motivation [2], and other terms [3] including; leisure reading, reading out-of-school, print exposure, voluntary reading, independent reading, reading habits, reading frequency, reading self-concept, and reading for pleasure.

**Fig 1:**
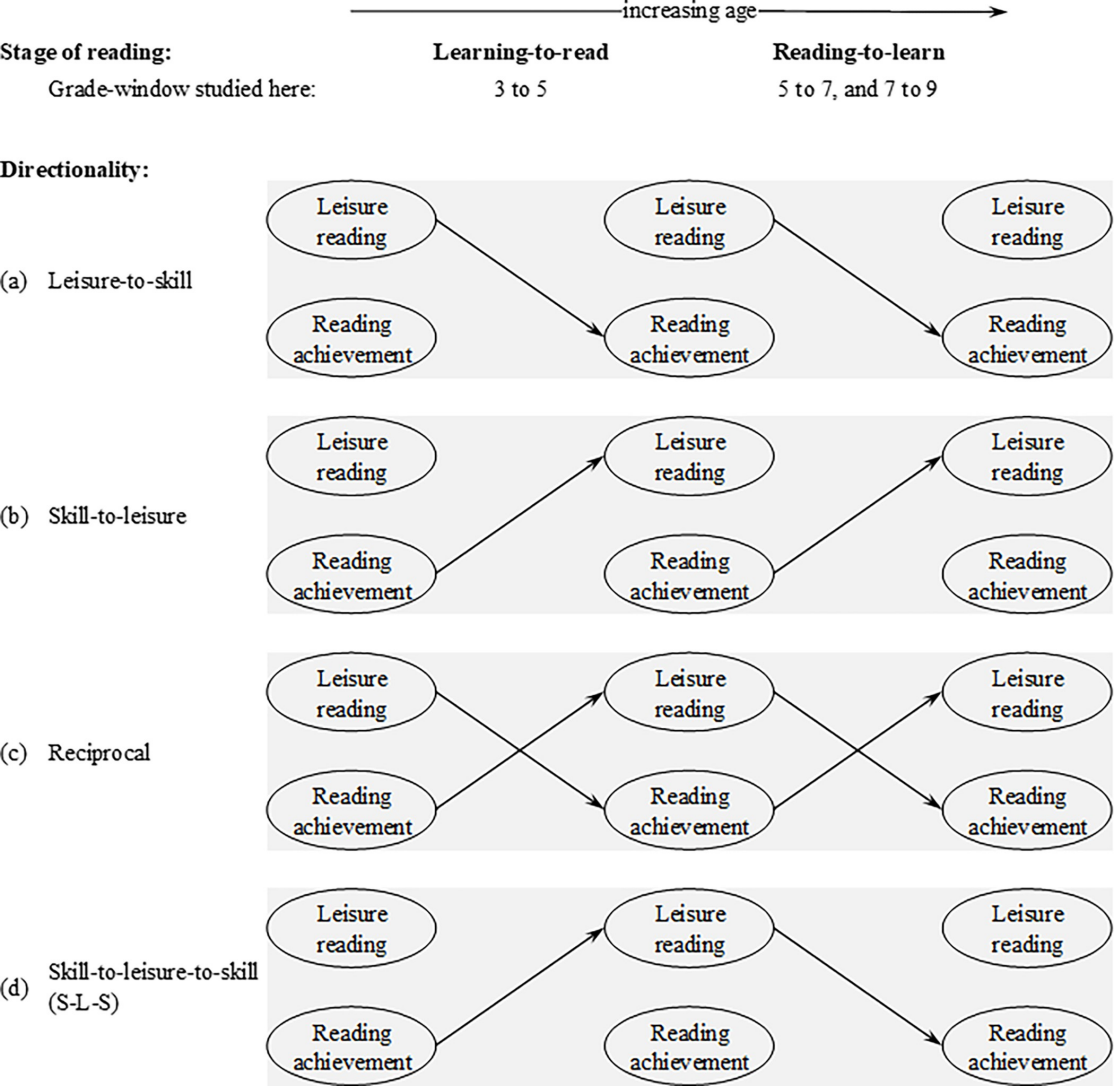
Different directionalities of the cross-paths (i.e. dashed paths in Fig 2) between reading achievement (i.e. skill) and leisure reading, depending on age, when modelling the within-person variance (grey circles in the middle of Fig 2).

The recent longitudinal evidence, which is detailed below, has produced two principal schools of thought. One is that achievement success leads to more leisure reading (*skill to leisure*; ‘*leisure*’ best represents the broader literature so is preferred; ‘*enjoy*’ represents the current study), irrespective of age (Fig 1, panel [b]). The other is that school achievement leads to more leisure reading (*skill to leisure*), but only within earlier reading stages. By late primary school and high school the directionality is reversed, with higher leisure reading leading to higher reading achievement (*leisure to skill*). Here, we label the latter school of thought the skill-to-leisure-to-skill directionality (S-L-S), as shown in Fig 1, panel (c).

The support for each school of thought possibly depends on the method of analysis. In turn, we consider the cross-lagged panel model (CLPM; longitudinal data), direction of causation models (DOC; twin data), and the more contemporary random-intercept cross-lagged panel model (RI-CLPM; longitudinal data). Our review of the CLPM suggests achievement influences leisure reading more so than the reverse, irrespective of age. Cross-lagged paths predict either achievement from the preceding time point of leisure, or vice versa, leisure from the preceding timepoint of achievement. For instance, reading achievement has been shown across multiple studies to predict a) reading pleasure/frequency (marginally) across grades 2 to 6 [4]; b) independent reading in ages 10 and 11 [5]; c) stronger reading habits than the reverse (*habits to achievement*) across grades 1 and 2 [6]; and d) motivation to read across grades 3 and 4, with some effects in the reverse direction [7].

The DOC models have afforded an alternative to longitudinal data by using twins. Despite this, the DOC models support the same *skill to leisure* directionality found with the CLPMs. Additionally, they encompassed students in grades 1 [3], 5 [8], and 4 to 9 [9], and this directionality remained the same irrespective of the student’s grade. Hence, the literature so far suggests reading achievement possibly predicts subsequent leisure more so than the reverse, irrespective of age. That said, questions have been raised regarding the CLPM.

Despite the CLPM estimating cross-paths, which are much acclaimed in developmental psychology, it is now apparent this CLPM conflates within- and between-person sources of variance [10]. Between-person effects represent the trait-like, or time-invariant stability, which is captured by a latent factor with each manifest loading constrained to one. In the current study, this contained average scores (computed for each individual using their scores from multiple occasions). For instance, a student may ‘on average’ score high on achievement throughout primary school, relative to a lower-performing peer. By contrast, within-person effects are where an individual might score above or below their trait-level at a particular timepoint. As such, within-person effects are more transient. The consequence of the CLPM conflating these within and between effects is that these models ‘typically give rise to estimates that are difficult (or impossible) to interpret meaningfully’ (14: p.1187). For example, what appears in CLPMs as cross-lagged effects of one variable on the subsequent time points of another variable can be entirely accounted for by between-person effects [11].

**Fig 2:**
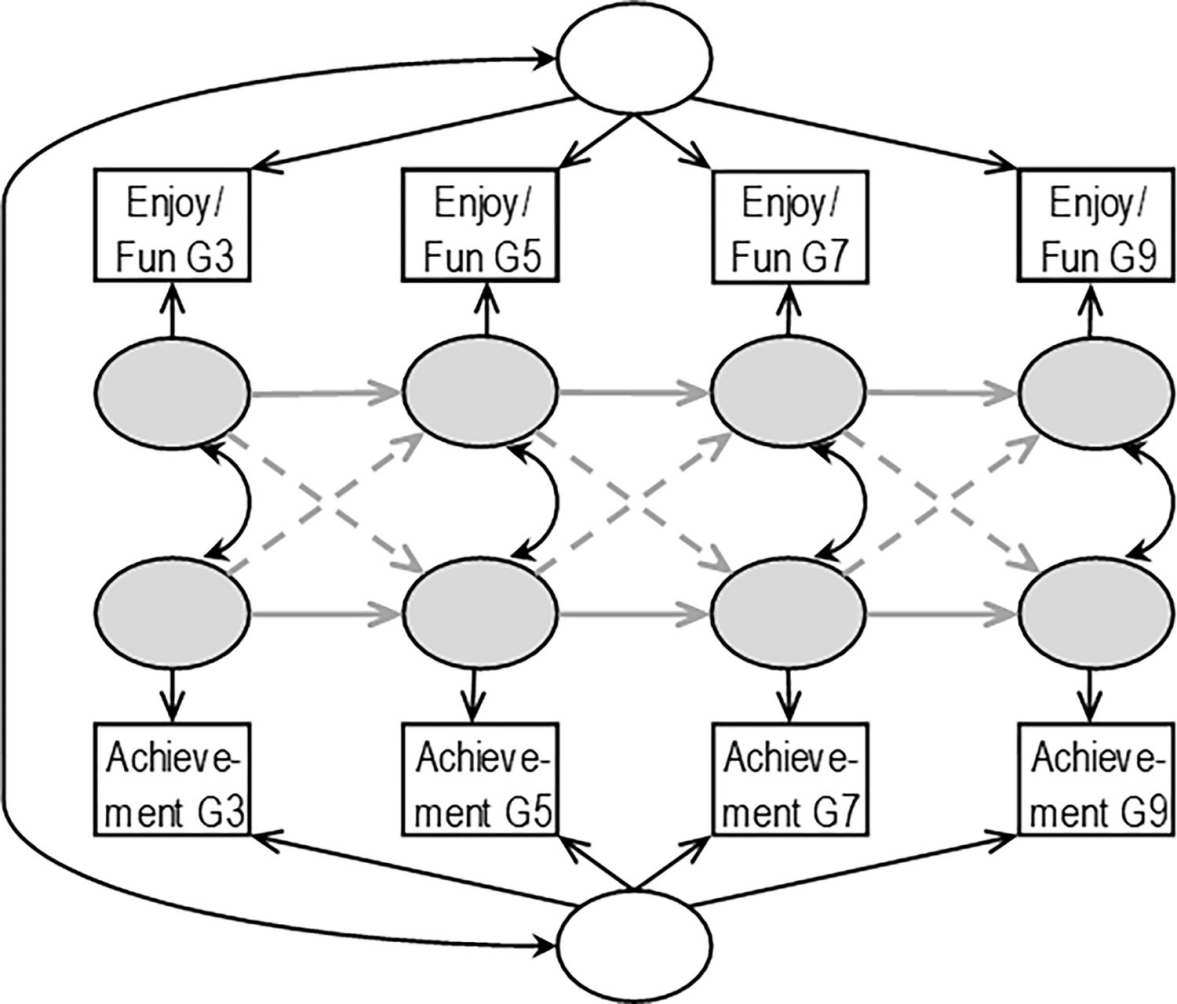
The random-intercept cross-lagged panel model (RI-CLPM) for the variables of the current study (reading enjoyment/fun and reading achievement) both measured at grades 3, 5, 7, and 9. Variance is partitioned into between-person latent variables (white circles) and within-person latent variables (grey circles), wherein all the residual variance not captured by the between-person latent variables is channelled into the within-person latent variables, which capture the time-specific effects of the model. The paths between the within-person latent variables comprise cross-sectional correlations (thin black lines), and cross-lagged coefficients (grey dotted lines), and auto-regressive coefficients (grey solid lines).

The random-intercept cross-lagged panel model (RI-CLPM, Fig 2) was designed to overcome this limitation of the CLPM. It separately estimates the between- and within-person sources of variance, thereby estimating cross-paths only in the within-person part of the model [10]. On a point of detail, whilst these cross-paths can contain between-person relations, these are only ever of the within-person deviations, or residuals, from the individual person average. As might be expected, differences between the cross-paths of the CLPM and RI-CLPM models are not unusual elsewhere in psychology. When re-modelled from the same data and using the RI-CLPM, former CLPM cross-paths disappeared [12,13], reversed [14], and appeared [12].

At this juncture, it is important to consider the potential economic benefit of segmenting the between and within portions of variance. Interventions and public policy arguably affect within-person effects more readily than between-person effects [15]. This is because within-person effects are mere variations around an individual’s mean and thus are more malleable. On between-person effects, there are compelling arguments for policy initiatives that change people’s average scores [15]. That said, these changes will typically not occur as readily. In fact, between-person effects are referred to as fixed effects in economics. Based on this reasoning, the more effective public policy levers will be based on and tailored to within-person effects where feasible. The least effective economic policies will be those that attempt to alter the between-person effects that have been inadvertently conflated with the within-person effects.

With regards to reading research, only two studies [8,16] have used the RI-CLPM to date, even when considering the broader ‘leisure’ literature. Interestingly, in these studies, the direction of the cross-paths depended on the student’s age. Specifically, in the early school years–prior to grade 4 –achievement predicted later leisure reading, while in the subsequent school years the directionality reversed. This flip in the effect’s direction was broadly consistent in both studies, even with distinct datasets. That said, Finnish samples were employed in both, so it remains to be seen if these results will be replicated in a different language and education system. Ultimately, the results of these two papers align with the aforementioned S-L-S school of thought. Hence, their findings lend further support to prior literature that has observed S-L-S in longitudinal data of reading self-concept [17], intrinsic motivation [18], and motivation more broadly, in a meta-analysis [19].

The S-L-S pattern is supported by theories on a) the changing stages of reading with age, b) the earlier stage (*skill to leisure*), and c) the later stage (*leisure to skill*). We discuss these in turn. First, Chall’s stage theory [20] asserts that reading increasingly becomes more difficult, rather than less, when the stage of instruction transitions from learning-to-read to reading-to-learn, which occurs by grade 4. These different stages are also defined by different skills and how readily those skills can be mastered [21]. The learning-to-read stage involves learning alphabet letters, phonics, and concepts of print. These are all skills that can be mastered and are, accordingly, ‘constrained’ skills. This stage also encompasses the next reading level: mastering phonemic awareness and fluency, which are ‘partially constrained’ skills that can be largely mastered, albeit more slowly with struggling students. At the other end, the later reading-to-learn stage involves instruction and individual differences in vocabulary and comprehension and, given that these are mastered to varying degrees, they represent reading skills that are ‘unconstrained’ [21].

In essence, the S-L-S occurs because at the earlier learning-to-read stage, the children that are better at these readily mastered skills will be subsequently more motivated to read and will enjoy it more–the *skill to leisure* directionality. By contrast, in the *leisure to skill* direction, children who have yet to master the constrained skills will be less driven by a leisure of reading to master them. It is the cycle from poor skills to limited enjoyment that manifests into the Matthew Effect, wherein the weaker students fall further behind over time on account of less leisure reading, while the stronger performing students have their skills subsequently reinforced by more leisure reading, which escalates into increasingly stronger achievement over time. There are claims that the Matthew Effect perpetuates throughout schooling, but the effects are arguably short lived, as is evidenced in the literature [22] and detailed below.

At the later reading stage this directionality appears to flip. The later stage is characterised by differences in the unconstrained skills of vocabulary and comprehension, as most students will have mastered the basics of reading and will be reading independently for comprehension. Hence, their enjoyment dictates variation in their reading frequency and material complexity, where the texts can become increasingly more challenging on account of these skills being unconstrained. This, in turn, influences their subsequent reading achievement (*enjoy to skill*). The *leisure to skill* direction is addressed by multiple theories. The expectancy-value theory [23] asserts that the child’s subjective value and expectations of success influence their achievement. Verbal efficiency theory [24] suggests that word-coding skills must be automatic and efficient in order to free up resources to attend to meaning and comprehension. Finally, [25] proposes a functional chain, wherein motivation increases reading volume, which in turn increases literacy.

This paper will assess the cross-lagged associations between achievement and enjoyment/fun across grades 3, 5, 7, and 9, thereby spanning the turning point of the S-L-S stages detailed above. Moreover, this study will be the first to use the RI-CLPM to specifically research reading ‘enjoyment’ and ‘fun’. In line with previous studies that also used the RI-CLPM, we hypothesize grade-dependent S-L-S directions of effect for the cross-lagged paths. In the mid-primary school years, we expect reading achievement will predict subsequent enjoyment/fun (*skill to enjoy*) more so than the reverse (*enjoy to skill*). In subsequent grades, on the other hand, reading enjoyment/fun will predict later achievement (*enjoy to skill*). We will assess this S-L-S hypothesis by considering the three possible directions, *skill to enjoy*, *enjoy to skill*, or reciprocal, within each reading stage: learning-to-read (the grade 3 to 5 window) and reading-to-learn (the grades 5 to 7 and 7 to 9 windows).

## Method

### Participants

The participants were from the Academic Development Study of Australian Twins (ADSAT; 26), which approached families registered with Twins Research Australia that had twins aged 8 to 16 years who were in grades 3, 5, 7, or 9 at Australian schools from 2008 to 2016. Recruitment was via mail to 8,604 parents a) listed with the Australian Twin Registry b) who had twin children that sat for the National Assessment Program: Literacy and Numeracy (NAPLAN) between 2008 and 2018. Of those 8,604 families, 2,824 agreed to participate–a 33% response rate–and were thereafter mailed/emailed a questionnaire bi-annually in grades 3, 5, 7 and/or 9. The NAPLAN results were obtained separately from state education departments. The research was approved by the University of New England Ethics Committee (‘A Twin Study of the NAPLAN’, approval numbers HE12-150 and HE18-163).

Demographics showed the sample was higher than the national average on socioeconomic status (SES) and NAPLAN [26], as expected for a volunteer sample. For instance, (a) postgraduate qualifications of the sample were higher than the population by 20% in the mothers and 12% in the fathers. (b) Eighty-two percent of the sample resided with both biological parents, up 10% on the national average. Finally, (c) the sample’s mean NAPLAN reading scores (with *s* in brackets) were 451 (88) and 615 (64) at grades 3 and 9 respectively; up on the national means of 420 (85) and 579 (66) at grades 3 and 9. The sample’s mean child age at grade 3 was 8.31 (0.35), just below the national average of 8.58, and was approximately two years older at each subsequent NAPLAN grade.

The analyses were of the 2,716 twin pairs (53% female) with data on at least one variable (4% missingness). This ensured sufficient power to detect small effects. To overcome the non-independence of twins, the individuals of each pair were randomly assigned as either twin-one or twin-two, with the analyses thereafter run separate for twins one and two. The ethnicity variable showed 96% of participants identified as European, 2% Asian, 1% Aboriginal Australian, and 1% other.

### Measures

#### Reading achievement

NAPLAN is an Australia-wide standardised school assessment that was introduced in 2008. It is arguably a test of both (a) achievement, by assessing current performance, and (b) aptitude, by assessing future academic potential; but we refer to it as achievement throughout. In grades 3, 5, 7, and 9, Australian school children sit tests in five domains: reading, spelling, grammar and punctuation, writing, and numeracy. Just reading is analysed here. The tests are administered nation-wide on the same three days in May each year. They are calibrated to be vertically and horizontally equated using Rasch modelling. Students receive an overall score between 1 and 1,000 in each of the five tests. That said, to ensure the RI-CLPMs converged we converted these scores to be on a similar scale to the reading for enjoyment/fun variables, which we achieved by dividing the NAPLAN scores by ten. The internal reliability of the grade 3 tests from 2008 to 2010 was .87 to .92 [27].

The NAPLAN achievement scores assess reading *comprehension* first and foremost. For the later grades, this will come as no surprise, but the test assesses *comprehension* rather than *fluency* or *phonemic awareness* even at grade 3. That said, at this early reading stage, *comprehension* and *fluency* are correlated [21], given that students are unable to comprehend text if they are unable to fluently read.

#### Reading enjoyment and reading for fun

The project administered a parent-report questionnaire consisting of 46 questions (detailed in 26) specific to each child in a twin pair, with a 75% to 80% response rate each year. Two of these questions were used in the present study: ‘How much do the children enjoy reading?’, and ‘How often do the children read for fun?’. Each question comprised a 7-point Likert-scale ranging from 1 = Not at all to 7 = Very much.

The correlations between reading enjoyment and reading for fun, with both measured at the same grade, were ~.70, ignoring slight differences at each grade. The test–retest correlations are presented in Table 1: over two years (grades 3–5, 5–7, and 7–9), these were higher for reading for enjoyment (~.75) than for reading for fun (~.65), and were similar to those of reading achievement scores (~.75).

**Table 1:** Means, Standard Deviations, correlations, and sample sizes of the variables analysed: Reported separately for twin-one (upper half) and twin-two (lower half).

					Reading enjoyment				Reading for fun				Reading achievement			
					G3	G5	G7	G9	G3	G5	G7	G9	G3	G5	G7	G9
Twin-one																
	N				1460	1546	1452	1272	1458	1545	1451	1269	1762	1944	1886	1644
	Mean				5.13	5.20	4.94	4.75	5.05	5.02	4.64	4.28	4.51	5.28	5.77	6.15
	Standard deviation				1.81	1.87	1.99	2.02	1.72	1.83	1.99	2.12	0.88	0.78	0.68	0.64
	Correlations															
		Reading enjoyment														
			G3		—											
			G5		.74	—										
			G7		.62	.77	—									
			G9		.55	.61	.80	—								
		Reading for fun														
			G3		.62	.49	.40	.40	—							
			G5		.52	.73	.59	.48	.64	—						
			G7		.47	.58	.75	.63	.50	.68	—					
			G9		.46	.49	.64	.78	.39	.50	.72	—				
		Reading achievement														
			G3		.52	.52	.48	.44	.37	.43	.38	.36	—			
			G5		.46	.51	.48	.39	.34	.43	.37	.34	.73	—		
			G7		.45	.44	.52	.47	.32	.36	.42	.40	.72	.76	—	
			G9		.46	.46	.49	.49	.30	.38	.39	.42	.68	.72	.78	—
Twin-two																
	N				1458	1540	1448	1269	1458	1540	1450	1269	1767	1944	1883	1641
	Mean				5.17	5.27	5.02	4.81	5.06	5.02	4.62	4.26	4.52	5.28	5.76	6.17
	Standard deviation				1.78	1.81	1.99	2.02	1.68	1.82	2.01	2.11	0.87	0.79	0.68	0.67
	Correlations															
		Reading enjoyment														
			G3		—											
			G5		.72	—										
			G7		.60	.77	—									
			G9		.53	.63	.78	—								
		Reading for fun														
			G3		.63	.52	.44	.41	—							
			G5		.52	.73	.59	.47	.64	—						
			G7		.48	.61	.77	.63	.50	.69	—					
			G9		.44	.50	.62	.78	.39	.49	.70	—				
		Reading achievement														
			G3		.48	.52	.45	.35	.34	.42	.38	.31	—			
			G5		.43	.51	.51	.43	.33	.45	.43	.34	.75	—		
			G7		.43	.49	.54	.47	.34	.42	.46	.40	.72	.74	—	
			G9		.43	.48	.53	.49	.35	.40	.45	.40	.71	.74	.79	—

Note: Correlation Ns were lowest between G3 and G9 and highest between G3 and G5 as follows; 548 and 1199 respectively for the enjoy-enjoy correlations, 840 and 1540 respectively for the achievement-achievement correlations, and 625 and 1221 respectively for the enjoy-achievement correlations (fun was similar to enjoy).

#### Covariates

The analyses covaried for sex (males = 0 and females = 1) and socioeconomic status (SES). SES was represented by a single factor score, as detailed in [22]. Among the five items included on the factor were the highest level of education and occupation reported by both parents and the family home’s neighbourhood SES. Occupation was scored on the International Socio-Economic Index of Occupational Status (ISEI) [28], while neighbourhood SES used the Index of Relative Socio-economic Advantage and Disadvantage [IRSAD; 29].

### Assumptions

There were no outliers on reading for enjoyment or fun. On reading achievement, we removed eight outlying scores based on a natural break in the data pattern. The outliers all had scores at a particular grade that were four or more standard deviations lower, but not higher, than the students’ own averages. This aligns with the logic that a student cannot cheat on a test and score substantially above their average, but if they do not put in effort they can score substantially below their average.

We screened histograms of each variable before checking the normality of the residuals. At all grades, the achievement variables were normal. The reading for enjoyment and fun variables were moderately negatively skewed at most grades, with the worst being enjoyment in grade 5 (z-skew = 13; percent per level: 4, 6, 9, 12, 15, 16, and 37) and the worst being fun in grade 3 (z-skew = 14; percent per level: 7, 3, 7, 12, 26, 24, and 22). The residuals, however, were normal and homoskedastic based on our most rigid test of this. Namely, multiple regressions between all grade-adjacent variables, such as grade 7 reading achievement, were predicted from grade 5 reading enjoyment and grade 5 reading achievement. Further, the large sample size and robust full information maximum likelihood (RFIML) estimator will minimise any bias from non-normality [30–32].

All bivariate associations were checked for linearity using scatterplots fitted with a Lowess line [33]. Linearity deviations were mild across scatterplots between the reading for enjoyment/fun and achievement variables, with the Lowess line typically curving away from linearity only at high achievement scores. These deviations will have served to marginally deflate the associations observed here. There was no multicollinearity.

### Missingness

The missingness by variables was lowest in grade 3, with 34% missing for reading achievement and 45% for the reading for enjoyment and fun variables. This is relative to the 2,824 individuals who agreed to participate in the study. The missingness by grade 9 was marginally higher (39% for achievement and 51% for the enjoyment/fun variables) primarily due to families moving during the study. The higher missingness on the reading for enjoyment and fun variables compared to achievement was due to a small number of families providing consent for us to access their achievement data from the state departments but then not responding to subsequent questionnaires that contained the enjoyment/fun variables (see 26).

On missingness by cases, there were fewer participants with four data waves. This was primarily due to participants either a) already being in grades 5, 7, or 9 when joining the project in the first year of NAPLAN (2008) when no NAPLAN testing was available to them for the earlier grades or b) having yet to sit their NAPLAN tests for grades 5, 7, or 9. Specifically, for the reading for enjoyment and fun there were 1,773 participants with two or more time points of data, 1,145 with more than three or more time points, and 507 with four time points of data (missingness of 37%, 59%, and 82% respectively, relative to those who agreed to participate). There were more participants for the NAPLAN (i.e. achievement) variables, as 2,232 had two or more time points of data, 1,560 had three or more time points, and 799 had four time points (missingness of 21%, 45% and 72%). It is reasonable to regard these data as missing at random (MAR), considering the primary reasons just noted. Because of the missingness, the RFIML estimator was used for all analyses, which handles both missing completely at random (MCAR) and MAR data [34].

### Statistical analyses

#### Which longitudinal model is optimal?

Is the RI-CLPM best suited for studying the cross-lagged relations between reading achievement and reading enjoyment/fun? Various models have been proposed for investigating whether reciprocal or prospective relationships exist in longitudinal data. One study [35] used ten different datasets to compare seven such models: the CLPM, the RI-CLPM, the autoregressive latent trajectory model (ALT), the latent curve model with structured residuals (LCM-SR), the bivariate latent change score model both with and without changes-to-changes extension, and the bivariate cross-lagged trait-state-error, also known as STARTS. Of these, the RI-CLPM was preferred since it consistently converged better than the alternatives. While [35] reported that the CLPM also fitted well, this claim has been questioned [36].

The following are further reasons to select the RI-CLPM over alternatives that model growth, such as the ALT. First, the goal in this research is to allow for time-specific effects (i.e. the paths were not equated across time), as these could potentially manifest as within-person variance as opposed to mere random noise. For instance, an environmental perturbation, such as transitioning from primary school to secondary school, could have flow-on effects of consistently poorer performance, which the RI-CLPM can capture. By contrast, growth models overlook within-person effects like this because they focus on between-person inference, and while a growth process represents a within-person change of a between-person effect, the slopes are ultimately a function of time as opposed to being time-specific [37]. Second, because there is no temporal order between the intercept and growth parameters, growth models are ‘not suitable for testing prospective effects between constructs’ [35]. Third, [38] considered the aforesaid models and built a unifying framework of their similarities and differences. They highlighted that the key point to consider when deciding between RI-CLPMs and growth models pertains to unobserved confounds and whether these are likely to be time-varying or time-unvarying. If such confounds are time-varying, such as the transition from primary to secondary school, then RI-CLPMs are more appropriate. By contrast, confounds that have a linear effect over time are better modelled with the ALT. Accordingly, the RI-CLPM is preferred here.

#### Fitting the RI-CLPMs

**Fig 3:**
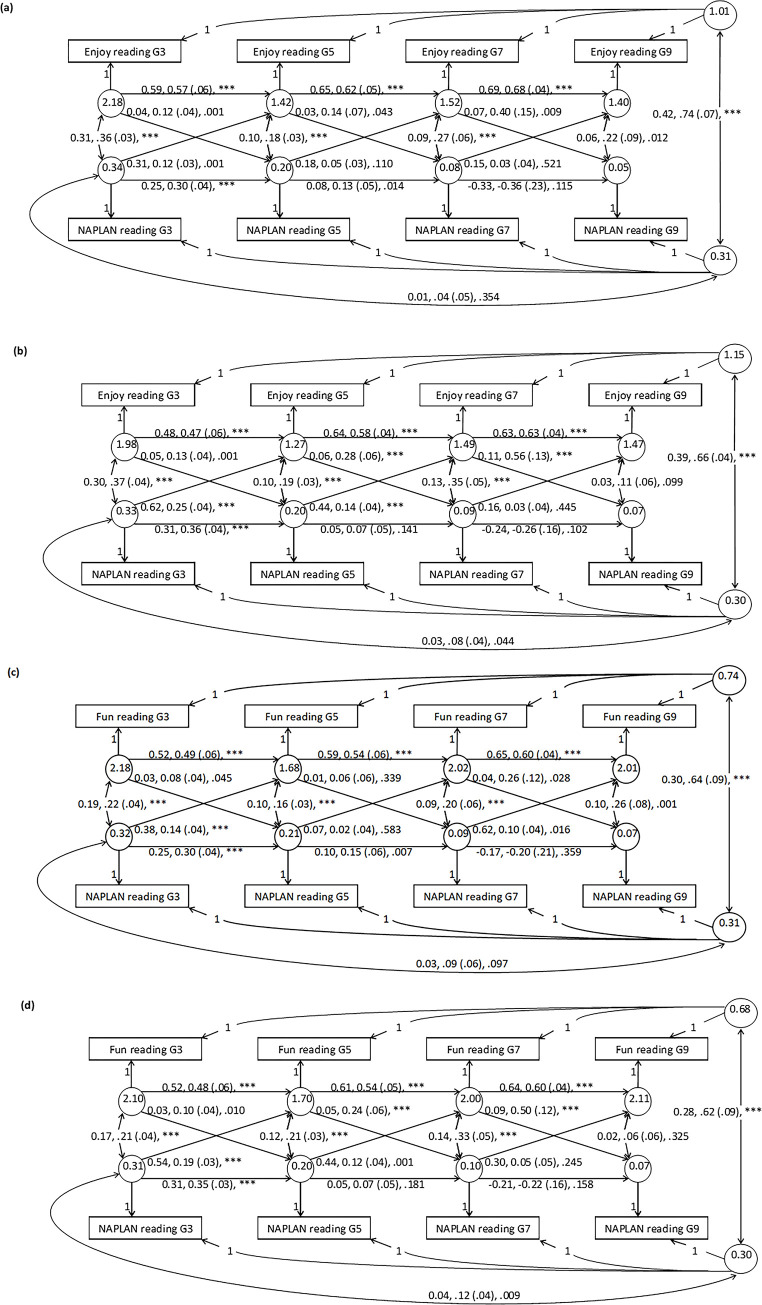
All parameter estimates of random-intercept cross-lagged panel models (RI-CLPMs) of reading enjoyment and reading achievement (NAPLAN) in twin-one [a] and twin-two [b], followed by reading for fun and reading achievement (NAPLAN) in twin-one [c] and twin-two [d]. The numbers reported on the single-headed-arrow paths are ordered as follows: Unstandardised betas, standardised betas, standard errors in brackets, and *p* (denoted as *** where *p* < .001). The same are reported for the double-headed-arrow paths, except the first two numbers represent covariances and correlations respectively. The unstandardized variances are reported within the circles of each latent variable. While covariates (sex and SES) were included in these analyses, they were omitted from in this figure to reduce clutter and are instead reported in the Results section.

RI-CLPMs were fitted to the unstandardized manifest variables of grades 3, 5, 7, and 9. All models were fitted twice: first for enjoyment-achievement and then for fun-achievement. Sex and SES were covaried out of the manifest variables, as demonstrated in [39]. Further, we augmented the standard structure of the RI-CLPM presented in Fig 2. According to a recent critique of the RI-CLPM [40], estimates will be unbiased only if the instruments modelled are in equilibrium, meaning their means and variances are constant across the timeframe modelled and in the window prior to and after the timeframe modelled. When instruments are not in equilibrium, a correlation between the first timepoint’s within-subject residual and the between-subject factor is added as a correction [40]. As shown in Table 1, reading achievement is not in equilibrium; hence, we applied this correlation correction to the RI-CLPMs (see Fig 3). Finally, we fitted the RI-CLPM without adding any constraints, in accordance with the typical practice in the literature and the advice of [35]. Mplus version 8.4 [41] was used for all analyses.

For the models to be identified, a minimum of three timepoints of data are needed. Our models are comfortably identified by being fitted to four time points. Further, the time points are equidistant, ensuring that we can interpret the estimates in a meaningful way [42].

## Results

Table 1 presents the descriptive statistics (M, σ, r, and N) of the variables analysed: reading for enjoyment/fun and reading achievement (i.e. NAPLAN). For all RI-CLPMs, the Supporting Information presents the Mplus scripts and outputs.

### Full (i.e. unconstrained) RI-CLPM

#### Model fits

The model fits (Table 2) were sound for the four RI-CLPM models (Fig 3): enjoyment–achievement and fun–achievement for both twins one and two. While the fun–achievement model differed from the saturated model in one twin (i.e. χ2 was significant), in the other twin and in the enjoyment–achievement models it did not. Our sizeable sample may have fuelled the single significant χ2. Accordingly, we made no attempt to resolve this by estimating more paths. The Tucker-Lewis Indices (TLI) and Bentler’s Comparative Fit Indices (CFI) were all above (a) .980 and (b) the benchmark of .950 [43], suggesting the models differ from models with covariances of zero. The Root Mean Squared Error of Approximations (RMSEA) and Standardized Root Mean Squared Residuals (SRMR) were below a) .033 and b) the benchmarks of .050 and .080, respectively [43]. These absolute fit indices suggested the models were sound. Finally, the residual correlations were all below .10, suggesting each model explained the data well [44].

**Table 2:** The model fit indices of four RI-CLPMs; enjoy-achievement and fun-achievement, both run for twins 1 and 2.

Variable		Model fit indices									
Twin no.		*χ* ^*2*^	*χ*^*2*^ *d*.*f*.	*χ*^*2*^ *p*	RMSEA	SRMR	CFI	TLI	AIC	BIC	BICa
Enjoy											
	1	19	8	.01320	.023	.018	.998	.992	31,218	31,525	31,360
	2	16	8	.04390	.019	.015	.999	.994	31,151	31,458	31,293
Fun											
	1	30	8	.00020	.032	.021	.997	.981	32,335	32,642	32,477
	2	11	8	.19610	.012	.014	1.000	.997	32,297	32,603	32,438

#### Between-person variance

The between-person latent variables–student average scores across grades 3, 5, 7 and 9 –were positively correlated (ranging *r* = .74 to .62, see Fig 3) between reading for enjoyment/fun and reading achievement, as expected. A large effect according to [45], is .51, and this result is greater. Likewise, one former paper [16] estimated a large effect, while another [8] did not, with a negligible estimate that was perhaps due to fewer time points across a wider timeframe.

#### Between- versus within-person variance

To gauge the relative variance contributions of the between and within latent variables, we averaged the four within latent variables and contrasted the unstandardised variances of the within and between latent variables. For the enjoyment/fun and achievement variables respectively, roughly two-thirds and one-third of the variance was captured by within-person effects, with between-person effects capturing the balance. This suggests the between-person variance was higher for the well-calibrated achievement variable than for the measures of enjoyment/fun, as expected.

#### Within-person variance

The within-person variance was passed into the cross-sectional correlations, cross-lagged coefficients, and auto-regressive coefficients, which we now review in turn.

#### Cross-sectional correlations

As expected, there were correlations between the sources of within-person variance of enjoyment/fun and achievement that were specific to the grades. Accordingly, students who enjoyed reading more (or less) than their own average at a particular grade also did better (or worse) than their own average on achievement at that grade. These occasion-specific correlations might reflect life circumstances at that particular grade that had a positive or negative influence on both variables of interest. These varied between .06 to .36 and were typically larger at grade 3 than grade 9. For the correlation at the first time point, benchmarks of small (.07), medium (.16), and large effect sizes (.38) exist [45], and these suggest the grade 3 correlations observed were large and medium effects for enjoy and fun respectively. These cross-sectional estimates aligned with those of the literature [8,16].

#### Cross-lagged paths

**Fig 4:**
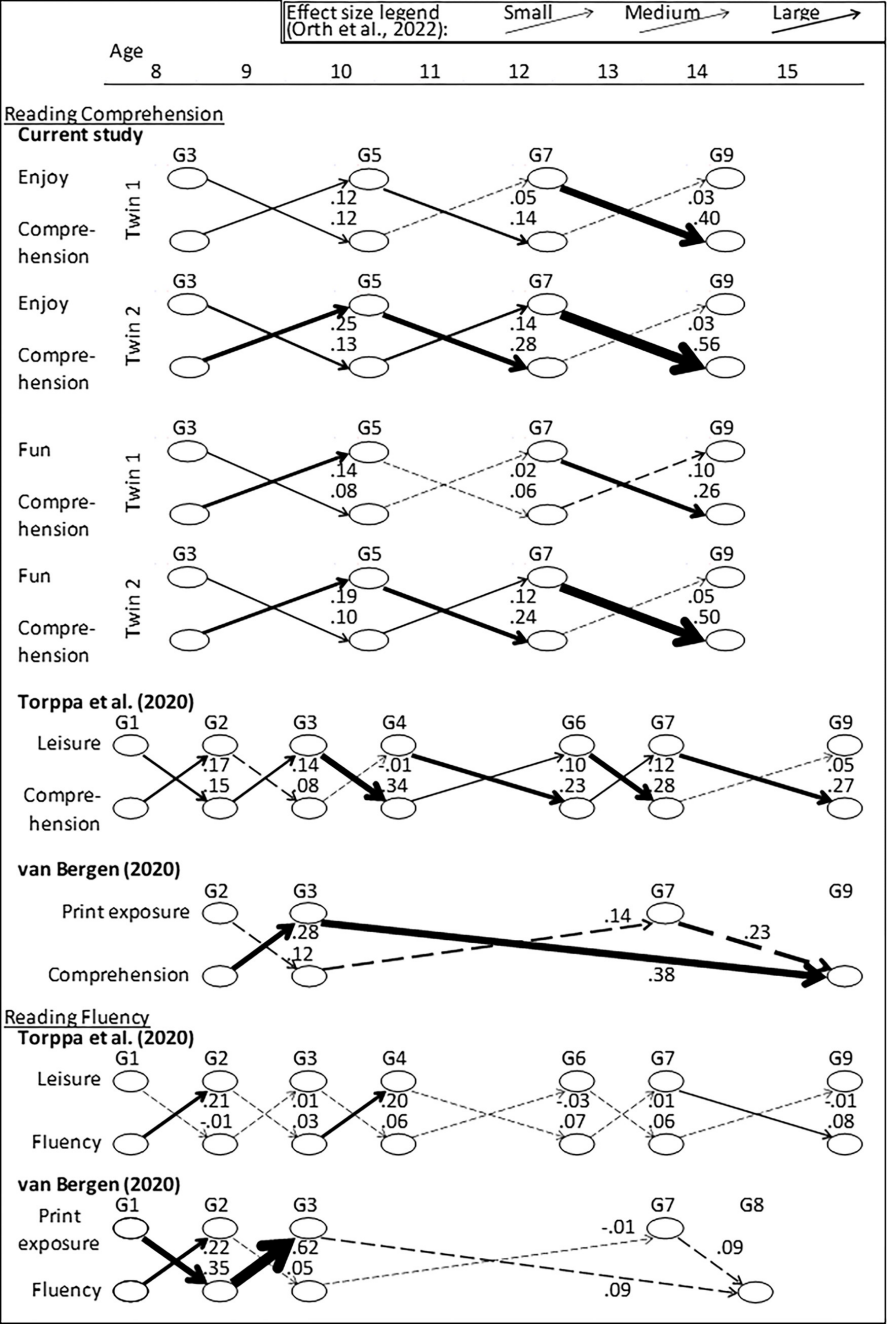
The line thickness illustrates the size of the standardized cross-lagged paths of all studies using RI-CLPMs of reading achievement and leisure reading (i.e. reading for enjoyment/fun in the case of the current study). Dashed lines are not significant at α = .05.

We interpret these coefficients relative to recently published small (.03), medium (.07), and large (.12) benchmark effect sizes of cross-lagged, standardised paths of RI-CLPM models [45]. These benchmark effect sizes are also presented in the legend at the top of Fig 4. This study’s results, presented in Fig 4, showed that all the significant paths (*α* < .05) had large effect sizes, while all the non-significant paths had small or medium effect sizes. Thus, we can infer these large effect sizes are likely greater than zero in the population of Australian school children studied in this paper.

All the cross-lagged paths were positive (see Fig 4), suggesting, for example, that high (or low) scores on earlier achievement tests (e.g. grade 3) seemed to infer subsequent higher (or lower) enjoyment/fun at later grades (e.g. grade 5). Furthermore, all cross-lagged effects are interpreted relative to each student’s average. For instance, where students scored higher on their grade 3 achievement than their average achievement reading level (i.e. calculated across the remaining grades 5, 7, and 9), parent reports suggest they enjoyed their reading at grade 5 more than their average reading enjoyment level.

To gauge whether the effect sizes (i.e. standardized betas) were larger in one direction (*skill to enjoy*) than the other (*enjoy to skill*), the line thickness in Fig 4 shows the magnitude of each cross-lagged path (top panel for enjoy-achievement, and second top panel for fun-achievement). In terms of nomenclature, when directionality is described as *skill to enjoy* or *enjoy to skill*, *“enjoy”* represents both reading enjoyment and reading for fun, while *“skill”* indicates reading achievement as measured by the achievement tests. Based on the findings, the directionality generally depended on the reading stage. In the earlier learning-to-read stage (grades 3 to 5), the betas of the *skill to enjoy* directionality were about twice those of the reverse, *enjoy to skill*. However, this was in only three of the four models (i.e. enjoy-achievement in twin-two, and fun-achievement in twin-one and twin-two); the fourth (enjoy-achievement in twin-one) supported reciprocal directionality. The twin-heterogeneity highlights that the *skill to enjoy* directionality was not ubiquitous at this early-stage–with some support for reciprocal directionality as well. Contrary to the early-stage, at the later reading-to-learn stage (grades 5 to 7 and 7 to 9) the general direction flipped, with the *enjoy to skill* directionality exceeding *skill to enjoy*, by two-fold for grades 5 to 7 and commonly by ten-fold for grades 7 to 9 (see Fig 4). This directional flip across the learning-to-read and reading-to-learn stages conforms with the S-L-S pattern hypothesised. We further discuss these cross-lagged effects relative to the previous literature in the Discussion session.

Notably, the standardized coefficients were particularly large for the grade 7 to 9 *enjoy to skill* cross-paths for twin-two in particular: .56 and .50 for enjoy to achievement and fun to achievement respectively. Relatedly, the error seemed higher in the last grade 7 to 9 window, which might account for these sizeable coefficients. First, the standard errors were higher in the last window (Fig 3). For grades 3 to 5, 5 to 7, and 7 to 9 respectively they were .04, .06, and .13 for the *enjoy to skill* cross-paths of twin-two, with the same occurring in twin-one and in the auto-regressive paths of achievement. Second, the variance (unstandardized) dropped for the within-subject residuals of achievement: 0.33, 0.20, 0.09, and 0.07 for twin-two, with the same occurring in twin-one (reported in the circles of the latent within-subject factors of Fig 3). In essence, these sizeable coefficients should be interpreted cautiously given they had larger standard errors and predicted outcomes with lower variance. Indeed, relative to typical RI-CLPM cross-paths they are potential outliers (see Fig 2 of 45). However, for this topic they are not unusual, with others reporting cross-paths of .34 (16), .35, and .62 (8) (see Fig 4).

#### Auto-regressive paths

Moving on from the cross-lagged paths, we now consider the larger auto-regressive effects (Fig 3). Unfortunately, unlike the aforementioned cross-lagged paths, there are no benchmarks of small, medium, or large effect sizes for these auto-regressive paths.

The auto-regressive effects were positive for the enjoy/fun variables, but they were both positive and negative for achievement. To explain, the standardised betas for the enjoy/fun variables were positive, large, and similar across time at roughly .50 to .60. Thus, less (or more) enjoyment/fun at any timepoint compounded into less (or more) enjoyment/fun at the subsequent time point. These estimates were comparable to those found in the literature [8,16]. However, unlike the enjoy/fun estimates, the betas for achievement were large but went from positive to negative. Hence, a student in grade 3 who performs worse (or better) than their average on reading achievement will also perform worse (or better) than their average in grade 5, with a standardised beta of around .30 to .35. On the other hand, if a student in grade 7 performs below (or above) their average, then by grade 9 the opposite occurs, and they will perform better (or worse), with a standardised betas of about -.20 to -.35.

These aforementioned results partially parallel those of the literature [8,16], where the estimates essentially declined but they did not become largely negative as they did in this study. Still, we have no reason to regard this large negative estimate as anomalous. In fact, it aligns with what is expected of a converging growth pattern of an ALT model, which has been observed for this variable using the same data [22].

#### Covariates

The covariates of sex and SES were modelled to predict the manifest variables of enjoy, fun, and achievement in grades 3, 5, 7, and 9 [39]. Since the sex and SES effects typically increased marginally across the grades, we report this range via the grades 3 and 9 standardized coefficients. Given that their results diverge, we first report sex, then SES. We report below on twin-one, but the results for twin-two were the same.

Sex typically predicted the enjoy (*p* < .001) and fun (*p* < .001) variables, but not achievement (*p* ranged from .002 to .220 and averaged .087). Positive coefficients indicated that the girls were higher, as follows. At grades 3 and 9, the sex to enjoy betas were *β* = 0.15 and *β* = 0.22 respectively, and the sex to fun betas were *β* = 0.09 and *β* = 0.18 respectively. By contrast, the sex to achievement betas were lower in grade 3 and 9, at *β* = .05 and .03 respectively. Furthermore, contrary to sex, SES predicted all the enjoy (*p* < .001), fun (*p* < .001), *and* achievement variables (*p* < .001). Based on the positive coefficients, higher SES was associated with higher scores on enjoy, fun and achievement. In grades 3 and 9, the *β* were 0.13 and 0.20 for enjoy, 0.15 and 0.20 for fun, and 0.32 and 0.38 for achievement respectively. Notably, the covariates did not dictate the essential findings of this paper, which remained the same when the covariates were omitted.

## Discussion

This study apportioned the association between reading for enjoyment/fun and reading achievement (i.e. NAPLAN) into the between- and within-person effects by using the RI-CLPM. Former research with the CLPM concluded that cross-lagged effects accounted for non-trivial portions of variance; however, since this encompassed between-person (trait-like) effects, it was unknown how much within-person variance would remain once the between-person variance was parsed out. Within-person effects accounted for a sizeable portion of the variance–half and one-third for the enjoyment and achievement variables, respectively. It remains to be seen whether this generous portion of within-person variance will replicate, and future research that uses multi-item scales, rather than the single items for enjoyment/fun used by this study, could plausibly find smaller effects.

Of this within-person variance, the cross-lagged paths were not incompatible with the S-L-S pattern across the learning-to-read and reading-to-learn stages [20], which is in line with what has been reported [8,16]. First, we discuss the earlier learning-to-read effects from achievement to subsequent enjoyment, followed by the later reading-to-learn effects from enjoyment to subsequent achievement. These influences represent the RI-CLPM’s time-specific effects. In the learning-to-read stage, achievement at grade 3 generally predicted enjoyment at grade 5 (*skill to enjoy*) more so than the reverse (*enjoy to skill*) at the same grades. This result has been aptly referred to [8,19] as the Matthew effect, wherein the ‘rich get richer’. Second, in the later reading-to-learn stage, the coefficients from reading enjoyment in grades 5 and 7 to reading achievement in grades 7 and 9 (*enjoy to skill*) exceeded those of the reverse (*skill to enjoy*). This reversing directionality across the reading stages supported the hypothesised S-L-S pattern.

Despite this support for S-L-S, it is crucial to note that this was not the only directional pattern to emerge. Ultimately, the pattern depended on whether we interpret the significant, the effect sizes (small, medium or large), or the relative effect sizes (the size of one coefficient compared to another)–all of which are alternative statistics for interpreting the same results. First, this study’s relative effect sizes mostly supported an S-L-S pattern, that is, when comparing how big the effect sizes were for the *skill to enjoy* versus the *enjoy to skill* directionality (top four panels of Fig 4). That said, other statistics showed support for S-L-S and reciprocal directionality. First, in the earlier learning-to-read stage, both directions (*skill to enjoy* and *enjoy to skill*) were significant and typically had large effect sizes (Fig 3)–supporting reciprocal directionality. Second, in the reading-to-learn stage, the significance and effect sizes supported a blend of *enjoy to skill* and reciprocal directionality during grades 5 to 7 at least; certainly by grades 7 to 9 these statistics consistently supported *enjoy to skill* (i.e. of S-L-S). This begs the question; which statistics are appropriate? While significance has been *the* traditional yardstick, some argue this practice should now be abandoned (while retaining *p*-values when interpreted on a continuum, rather than a cutoff) [46–48]. Instead, graphs of relative effects size are proposed as an alternative [49,50], among many [51], with Fig 4 depicting this study’s attempt to answer this call. Ultimately, this paper’s results were a confluence of evidence in favor of either reciprocity or S-L-S over any other pattern (see Fig 1).

The reciprocal directionality we observed aligned with prior literature [2]. That literature, however, predated the RI-CLPM. The only other studies to have used the RI-CLPM with broadly equivalent variables [8,16] paralleled the current study in failing to discount the S-L-S pattern. Accordingly, we now turn our attention to this pattern but acknowledge that the evidence for it, over reciprocity, is equivocal. Fig 4 illustrates the studies that have used the RI-CLPM: the current study (top four panels) and the previous literature [8,16] (lower panels). As shown, the reversing directionality across the learning-to-read and reading-to-learn stages is most evident when the standardized betas of the cross-lagged paths are weighted by line thickness.

In interpreting Fig 4, it is important to understand how the instruments used to measure reading in these studies–fluency and comprehension–vary in their effectiveness at different stages of reading. Comprehension measures the ability to interpret meaning from text. Further, it is an effective instrument for measuring reading at the later reading-to-learn stage, when students have largely mastered reading [21]. Earlier on, however, when students are still learning to read, fluency (essentially the number of words read per minute) is often used to capture reading variance. The problem with fluency, however, is that the vast majority of students are fluent readers by grade 4, creating a ceiling effect where most students get high scores, which serves to create a distribution with a pronounced negative skew [21]. Fluency, therefore, is less effective at capturing reading variance than comprehension in later primary school and high school. Further, the two instruments are uncorrelated at the latter grades [21]. By contrast, at the earlier grades fluency and comprehension are correlated for the simple fact that fluency is necessary for comprehension. Accordingly, students who are fluent will be better able to comprehend text, and those who are not will comprehend less. As a consequence, fluency and comprehension are correlated in the earlier years, and both are effective at capturing reading variance, with the predominance of the fluency instrument over comprehension perhaps being greater the lower the grade. Therefore, to interpret Fig 4, it is important to give weight to fluency (bottom two panels) and, to some extent, comprehension (top six panels) in the earlier grades. By contrast, in the later grades most interpretive weight should be given to the effects of comprehension (top six panels).

As Fig 4 illustrates, while [8,16] ran RI-CLPMs separately for comprehension (middle two panels of Fig 4) and fluency (the bottom two panels), the current study (the top four panels) only had access to a measure of comprehension. However, given that comprehension is correlated with fluency in the earlier years, it is no surprise that the effects of the current study are analogous with those of fluency in [8,16], at least in the early years. By contrast, in the later grades the fluency effects in both [8,16] become small and not significant and depart from those of comprehension.

The S-L-S pattern is more apparent across the studies of Fig 4 when the acute lens of fluency/comprehension effectiveness at different stages is added. At earlier grades, the greater directionality from *skill to leisure*, compared to either the reverse or reciprocal directionality, is more apparent for the fluency instruments (bottom of Fig 4), but is also partly apparent for comprehension. That said, at these earlier grades the directionality is not always consistent, but there is more support for the *skill to leisure* direction than the alternatives.

At the later grades, when considering comprehension, a directionality from *leisure to skill* is apparent (top six panels of Fig 4), while for fluency, which is uncorrelated with comprehension at these later grades, there are few effects at all. Collectively, the studies to date using the RI-CLPM fail to discount the S-L-S pattern. This is despite the studies comprising data from Finland [8,16] and Australia (the current study).

Regarding the later reading-to-learn stage, the pattern of these RI-CLPM studies, from *leisure to skill*, contradicted the findings of the direction of causation (DoC) models of twin studies [9,52], which both suggested the opposite *skill to leisure* directionality. This disparity could result from slightly different instruments. Specifically, literacy achievement (a composite of reading, spelling, and writing) was modelled in [52] rather than reading achievement. That said, the instruments of [9] did correspond with the three RI-CLPM papers (the current study, 8,16). Alternatively, the disparity could stem from the RI-CLPM or the DoC models, and on the latter, we simply acknowledge that [52] give one plausible explanation:

‘It may be that something about the twin direction-of-causation method makes finding a unidirectional causal relation more likely, especially above a bidirectional relation, which costs an extra degree of freedom in the model.’

### Generalisability

Our findings may be sample-specific for two reasons. First, we used a sample of twins who are unusual in that they compete against their own twin. It is possible the time-specific effects we observed are specific to twins. That said, for achievement at least, we had the sibling data. By running a random-intercept model, which is ‘half’ a RI-CLPM (i.e., for just achievement reading and not reading enjoyment as well), we were able to check the twin results generalised to their non-twin siblings. This resulted in effect sizes consistent in their direction and magnitude (i.e. small, medium, or large). That said, we welcome attempts to replicate these findings in non-twin samples. Second, our study’s volunteers were one-third of those approached, representing only 15% of the twins registered with Twins Research Australia. It is no surprise that our sample was slightly above the Australian average on SES [26]. However, we covaried out SES, and even when we dropped this covariate from the model (results not shown), the conclusions were the same. Hence, it seems unlikely this sampling bias, at least, will result in the findings not generalising beyond our sample.

#### Limitations

Aside from the above-mentioned matters, our findings could also be limited by the long window between measurement occasions, which was two years–the standard time-interval of the national NAPLAN tests. That said, cross-lagged influences arguably dissipate over time. Accordingly, while our two-year window might have missed effects occurring within a shorter timeframe, we did observe effects across the two-year window, which may underestimate the effects of shorter timeframes [53]. Equally, despite anticipating effects across a shorter timescale (daily, fortnightly, three-monthly, or even annually), cross-lagged effects were only evidence on the longer biennial timescale, according to a recent RI-CLPM of depression and parent support [54]. Another limitation is we did not model the classroom and school-level variance, and to the extent these represent systematic rather than unsystematic effects, they will have biased the reported results. Finally, we made few attempts to detect unobserved confounds but regard this as an important avenue for future research.

### Implications

If the time-specific effects observed in this study are a) genuine deviations from a within-person average, which could plausibly be cultivated in the same way via intervention, and b) no mediating variables exist, which is highly unlikely [55], then these effects can potentially improve reading achievement in the middle years of schooling. If the evidence favoring S-L-S over a reciprocal directionality becomes less equivocal than is presently the case, it will have the following implications. While all school years are likely to be important when it comes to cultivating an intrinsic interest in leisure reading, the final years of primary school and beginning years of high school may represent the optimal window for this. In turn, education policy could be adopted to target the ideal ages and subjects for deploying costly yet necessary resources.

#### Future research

Assuming the pronounced effects observed here continue to stand up to replication, then it is likely there are mediators or even moderators of these time-specific effects, and such future research would be fruitful.

## Conclusion

In studying the effects of within-person variance of reading across grades 3, 5, 7, and 9, this study found no grounds to discount the S-L-S hypothesis. Further, this pattern corresponded to the findings of the only other authors who had fitted RI-CLPMs to equivalent variables. That said, we could not discount the reciprocal directionality either. To this end, twin heterogeneity highlighted the frailty of support for one directionality over the other. Given twins share family environments, and genes (to 75% on average across the blend of monozygotic and dizygotic twins used here), the observed heterogeneity is expected to be yet greater yet again for independent samples of the same size. Ultimately, the current study is but a single sample. It needs to be replicated and scrutinised with alternate datasets that ultimately encompass different languages and education systems as well as longitudinal data with biannual or annual assessments throughout the primary and secondary school years.

## Supporting information

S1 File The supporting information below presents, in order, the Mplus scripts and outputs of the following RI-CLPMs:
A) Reading enjoyment and reading achievement of twin 1,
B) Reading for fun and reading achievement of twin 1,
C) Reading enjoyment and reading achievement of twin 2, and
D) Reading for fun and reading achievement of twin 2.
(DOCX)
