# Supplementary material for: Do student differences in reading enjoyment relate to achievement when using the random-intercept cross-lagged panel model across primary and secondary school?
Source: PLoS One. 2023 Jun 9;18(6):e0285739. doi: 10.1371/journal.pone.0285739 (PMC10256200; doi:10.1371/journal.pone.0285739)
Supplement: S1 File — The supporting information below presents, in order, the Mplus scripts and outputs of the following RI-CLPMs: A) Reading enjoyment and reading achievement of twin 1, B) Reading for fun and reading achievement of twin 1, C) Reading enjoyment and reading achievement of twin 2, and D) Reading for fun and reading achievement of twin 2. (DOCX) [file pone.0285739.s001.docx]

**Supporting Information**

A. Reading enjoyment and reading achievement of twin 1

Mplus VERSION 8.7

MUTHEN & MUTHEN

03/30/2023 3:13 PM

INPUT INSTRUCTIONS

TITLE: sex ses rE1-rE4 rF1-rF4 rN1-rN4 mE1-mE4 mP1-mP4 mG1-mG4 mN1-mN4

DATA: FILE IS 4.4Tw1noDssAllData.dat;

VARIABLE: NAMES ARE sex ses X1-X4 rF1-rF4 Y1-Y4 mE1-mE4 mP1-mP4 mG1-mG4 mN1-mN4;

USEVARIABLES ARE sex ses X1 X2 X3 X4 Y1 Y2 Y3 Y4 ;

MISSING ARE ALL (-999);

ANALYSIS: Estimator=MLR ;

ANALYSIS: MODEL=NOCOV; !Exog vars correlated by default, but this fixes ALL corrs=0. Use W

MODEL:

!___Intercept factor !___BY statements

ix BY x1@1 x2@1 x3@1 x4@1;

iy BY y1@1 y2@1 y3@1 y4@1;

!___Covariates

x1 x2 x3 x4 y1 y2 y3 y4 ON sex;

x1 x2 x3 x4 y1 y2 y3 y4 ON ses;

!___Latent variables

Wx1 BY x1@1;

Wx2 BY x2@1;

Wx3 BY x3@1;

Wx4 BY x4@1;

Wy1 BY y1@1;

Wy2 BY y2@1;

Wy3 BY y3@1;

Wy4 BY y4@1;

!___Autoregressions !___ON statements

Wx2 ON Wx1;

Wx3 ON Wx2;

Wx4 ON Wx3;

Wy2 ON Wy1;

Wy3 ON Wy2;

Wy4 ON Wy3;

!___Crosslagged paths

Wy2 ON Wx1;

Wy3 ON Wx2;

Wy4 ON Wx3;

Wx2 ON Wy1;

Wx3 ON Wy2;

Wx4 ON Wy3;

!__VARIANCE STRUCTURE:

!____Variables estimated/fixed

x1-x4@0;

y1-y4@0;

Wx1-Wx4;

Wy1-Wy4;

ix;

iy;

!____WITH statements

ix WITH iy;

Wx1 WITH Wy1;

Wx2 WITH Wy2;

Wx3 WITH Wy3;

Wx4 WITH Wy4;

!ix WITH Wx1;

iy WITH Wy1;

!__MEAN STRUCTURE: !____Variables estimated/fixed

!results identical if below 2 lines are omitted (as model seems to estimate manifest-me

! but here i explicitly estimate them (so is visible)!

[x1-x4];

[y1-y4];

!results same if below 2 lines are omitted, but adding in residual-means is no cost to

[Wx1-Wx4@0];

[Wy1-Wy4@0];

!do not estimate below means, model becomes unidentified despite indentical vars n cova

! Hence, means below are fixed at zero, or omit these 2 lines entirely and results will

[ix @0];

[iy @0];

OUTPUT: TECH1 TECH4 STDYX SAMPSTAT RESIDUAL;

*** WARNING

Input line exceeded 90 characters. Some input may be truncated.

ANALYSIS: MODEL=NOCOV; !Exog vars correlated by default, but this fixes ALL corrs=0. Use WI

*** WARNING

Input line exceeded 90 characters. Some input may be truncated.

!results identical if below 2 lines are omitted (as model seems to estimate manifest-mea

*** WARNING

Input line exceeded 90 characters. Some input may be truncated.

!results same if below 2 lines are omitted, but adding in residual-means is no cost to m

*** WARNING

Input line exceeded 90 characters. Some input may be truncated.

!do not estimate below means, model becomes unidentified despite indentical vars n covar

*** WARNING

Data set contains cases with missing on all variables.

These cases were not included in the analysis.

Number of cases with missing on all variables: 2

*** WARNING

Data set contains cases with missing on x-variables.

These cases were not included in the analysis.

Number of cases with missing on x-variables: 7

6 WARNING(S) FOUND IN THE INPUT INSTRUCTIONS

sex ses rE1-rE4 rF1-rF4 rN1-rN4 mE1-mE4 mP1-mP4 mG1-mG4 mN1-mN4

SUMMARY OF ANALYSIS

Number of groups 1

Number of observations 2695

Number of dependent variables 8

Number of independent variables 2

Number of continuous latent variables 10

Observed dependent variables

Continuous

X1 X2 X3 X4 Y1 Y2

Y3 Y4

Observed independent variables

SEX SES

Continuous latent variables

IX IY WX1 WX2 WX3 WX4

WY1 WY2 WY3 WY4

Estimator MLR

Information matrix OBSERVED

Maximum number of iterations 1000

Convergence criterion 0.500D-04

Maximum number of steepest descent iterations 20

Maximum number of iterations for H1 2000

Convergence criterion for H1 0.100D-03

Input data file(s)

4.4Tw1noDssAllData.dat

Input data format FREE

SUMMARY OF DATA

Number of missing data patterns 126

COVARIANCE COVERAGE OF DATA

Minimum covariance coverage value 0.100

PROPORTION OF DATA PRESENT

Covariance Coverage

X1 X2 X3 X4 Y1

________ ________ ________ ________ ________

X1 0.540

X2 0.444 0.571

X3 0.323 0.423 0.537

X4 0.203 0.301 0.383 0.471

Y1 0.507 0.452 0.339 0.226 0.650

Y2 0.449 0.529 0.429 0.319 0.568

Y3 0.347 0.437 0.492 0.393 0.448

Y4 0.232 0.327 0.393 0.426 0.310

SEX 0.540 0.571 0.537 0.471 0.650

SES 0.540 0.571 0.537 0.471 0.650

Covariance Coverage

Y2 Y3 Y4 SEX SES

________ ________ ________ ________ ________

Y2 0.718

Y3 0.580 0.696

Y4 0.439 0.535 0.607

SEX 0.718 0.696 0.607 1.000

SES 0.718 0.696 0.607 1.000 1.000

SAMPLE STATISTICS

ESTIMATED SAMPLE STATISTICS

Means

X1 X2 X3 X4 Y1

________ ________ ________ ________ ________

5.016 5.160 4.935 4.708 4.492

Means

Y2 Y3 Y4 SEX SES

________ ________ ________ ________ ________

5.299 5.786 6.159 1.507 0.016

Covariances

X1 X2 X3 X4 Y1

________ ________ ________ ________ ________

X1 3.352

X2 2.569 3.529

X3 2.198 2.803 3.864

X4 1.969 2.256 3.107 3.996

Y1 0.812 0.811 0.794 0.701 0.748

Y2 0.647 0.728 0.717 0.611 0.487

Y3 0.542 0.582 0.676 0.620 0.417

Y4 0.518 0.572 0.640 0.638 0.379

SEX 0.140 0.149 0.172 0.223 0.024

SES 0.279 0.352 0.380 0.412 0.260

Covariances

Y2 Y3 Y4 SEX SES

________ ________ ________ ________ ________

Y2 0.609

Y3 0.400 0.463

Y4 0.368 0.349 0.423

SEX 0.012 0.000 0.008 0.250

SES 0.247 0.247 0.231 -0.008 1.098

Correlations

X1 X2 X3 X4 Y1

________ ________ ________ ________ ________

X1 1.000

X2 0.747 1.000

X3 0.611 0.759 1.000

X4 0.538 0.601 0.791 1.000

Y1 0.513 0.500 0.467 0.405 1.000

Y2 0.453 0.497 0.468 0.392 0.723

Y3 0.435 0.456 0.506 0.456 0.708

Y4 0.435 0.468 0.501 0.491 0.674

SEX 0.153 0.158 0.175 0.224 0.056

SES 0.146 0.179 0.185 0.197 0.287

Correlations

Y2 Y3 Y4 SEX SES

________ ________ ________ ________ ________

Y2 1.000

Y3 0.753 1.000

Y4 0.726 0.789 1.000

SEX 0.032 -0.001 0.025 1.000

SES 0.303 0.347 0.338 -0.016 1.000

MAXIMUM LOG-LIKELIHOOD VALUE FOR THE UNRESTRICTED (H1) MODEL IS -21452.104

UNIVARIATE SAMPLE STATISTICS

UNIVARIATE HIGHER-ORDER MOMENT DESCRIPTIVE STATISTICS

Variable/ Mean/ Skewness/ Minimum/ % with Percentiles

Sample Size Variance Kurtosis Maximum Min/Max 20%/60% 40%/80% Median

X1 5.124 -0.686 1.000 4.67% 3.000 5.000 6.000

1455.000 3.269 -0.599 7.000 31.82% 6.000 7.000

X2 5.197 -0.719 1.000 4.87% 3.000 5.000 6.000

1540.000 3.522 -0.674 7.000 37.99% 6.000 7.000

X3 4.945 -0.553 1.000 7.61% 3.000 5.000 5.000

1446.000 3.958 -0.950 7.000 34.51% 6.000 7.000

X4 4.751 -0.471 1.000 9.62% 3.000 4.000 5.000

1268.000 4.072 -1.035 7.000 28.71% 6.000 7.000

Y1 4.506 0.005 1.932 0.06% 3.780 4.309 4.523

1752.000 0.766 -0.036 7.600 0.06% 4.740 5.230

Y2 5.277 0.027 2.657 0.05% 4.647 5.100 5.253

1934.000 0.608 0.455 8.420 0.05% 5.480 5.929

Y3 5.770 0.083 3.662 0.05% 5.184 5.590 5.750

1876.000 0.455 -0.032 8.626 0.05% 5.928 6.338

Y4 6.149 -0.058 4.094 0.06% 5.623 6.000 6.150

1635.000 0.411 0.331 9.075 0.06% 6.294 6.670

SEX 1.507 -0.029 1.000 49.28% 1.000 1.000 2.000

2695.000 0.250 -1.999 2.000 50.72% 2.000 2.000

SES 0.016 0.131 -2.823 0.04% -0.887 -0.358 -0.056

2695.000 1.098 -0.569 2.935 0.04% 0.248 0.987

THE MODEL ESTIMATION TERMINATED NORMALLY

MODEL FIT INFORMATION

Number of Free Parameters 52

Loglikelihood

H0 Value -15557.148

H0 Scaling Correction Factor 1.0851

for MLR

H1 Value -15546.609

H1 Scaling Correction Factor 1.0858

for MLR

Information Criteria

Akaike (AIC) 31218.295

Bayesian (BIC) 31525.051

Sample-Size Adjusted BIC 31359.831

(n* = (n + 2) / 24)

Chi-Square Test of Model Fit

Value 19.330*

Degrees of Freedom 8

P-Value 0.0132

Scaling Correction Factor 1.0904

for MLR

* The chi-square value for MLM, MLMV, MLR, ULSMV, WLSM and WLSMV cannot be used

for chi-square difference testing in the regular way. MLM, MLR and WLSM

chi-square difference testing is described on the Mplus website. MLMV, WLSMV,

and ULSMV difference testing is done using the DIFFTEST option.

RMSEA (Root Mean Square Error Of Approximation)

Estimate 0.023

90 Percent C.I. 0.010 0.036

Probability RMSEA <= .05 1.000

CFI/TLI

CFI 0.998

TLI 0.992

Chi-Square Test of Model Fit for the Baseline Model

Value 7512.222

Degrees of Freedom 44

P-Value 0.0000

SRMR (Standardized Root Mean Square Residual)

Value 0.018

MODEL RESULTS

Two-Tailed

Estimate S.E. Est./S.E. P-Value

IX BY

X1 1.000 0.000 999.000 999.000

X2 1.000 0.000 999.000 999.000

X3 1.000 0.000 999.000 999.000

X4 1.000 0.000 999.000 999.000

IY BY

Y1 1.000 0.000 999.000 999.000

Y2 1.000 0.000 999.000 999.000

Y3 1.000 0.000 999.000 999.000

Y4 1.000 0.000 999.000 999.000

WX1 BY

X1 1.000 0.000 999.000 999.000

WX2 BY

X2 1.000 0.000 999.000 999.000

WX3 BY

X3 1.000 0.000 999.000 999.000

WX4 BY

X4 1.000 0.000 999.000 999.000

WY1 BY

Y1 1.000 0.000 999.000 999.000

WY2 BY

Y2 1.000 0.000 999.000 999.000

WY3 BY

Y3 1.000 0.000 999.000 999.000

WY4 BY

Y4 1.000 0.000 999.000 999.000

WX2 ON

WX1 0.591 0.055 10.693 0.000

WY1 0.306 0.084 3.655 0.000

WX3 ON

WX2 0.650 0.046 14.022 0.000

WY2 0.176 0.109 1.612 0.107

WX4 ON

WX3 0.692 0.039 17.578 0.000

WY3 0.152 0.239 0.635 0.526

WY2 ON

WY1 0.248 0.031 8.083 0.000

WX1 0.039 0.012 3.293 0.001

WY3 ON

WY2 0.075 0.035 2.166 0.030

WX2 0.026 0.012 2.133 0.033

WY4 ON

WY3 -0.326 0.207 -1.574 0.115

WX3 0.065 0.022 2.924 0.003

X1 ON

SEX 0.569 0.085 6.710 0.000

SES 0.261 0.040 6.543 0.000

X2 ON

SEX 0.605 0.083 7.271 0.000

SES 0.324 0.039 8.303 0.000

X3 ON

SEX 0.703 0.087 8.035 0.000

SES 0.351 0.041 8.633 0.000

X4 ON

SEX 0.907 0.094 9.641 0.000

SES 0.381 0.044 8.606 0.000

Y1 ON

SEX 0.103 0.036 2.866 0.004

SES 0.238 0.017 13.658 0.000

Y2 ON

SEX 0.057 0.031 1.814 0.070

SES 0.225 0.015 15.107 0.000

Y3 ON

SEX 0.006 0.027 0.235 0.814

SES 0.225 0.013 16.980 0.000

Y4 ON

SEX 0.040 0.027 1.473 0.141

SES 0.211 0.013 16.263 0.000

IX WITH

IY 0.417 0.038 11.087 0.000

WX1 WITH

WY1 0.311 0.040 7.700 0.000

WX2 WITH

WY2 0.096 0.018 5.210 0.000

WX3 WITH

WY3 0.093 0.021 4.395 0.000

WX4 WITH

WY4 0.060 0.022 2.763 0.006

IY WITH

WY1 0.014 0.015 0.957 0.338

Means

IX 0.000 0.000 999.000 999.000

IY 0.000 0.000 999.000 999.000

WX1 0.000 0.000 999.000 999.000

WY1 0.000 0.000 999.000 999.000

Intercepts

X1 4.149 0.139 29.750 0.000

X2 4.245 0.138 30.758 0.000

X3 3.870 0.144 26.908 0.000

X4 3.334 0.152 21.916 0.000

Y1 4.332 0.058 74.959 0.000

Y2 5.210 0.050 104.375 0.000

Y3 5.773 0.043 132.708 0.000

Y4 6.096 0.044 140.108 0.000

WX2 0.000 0.000 999.000 999.000

WX3 0.000 0.000 999.000 999.000

WX4 0.000 0.000 999.000 999.000

WY2 0.000 0.000 999.000 999.000

WY3 0.000 0.000 999.000 999.000

WY4 0.000 0.000 999.000 999.000

Variances

IX 1.006 0.257 3.916 0.000

IY 0.313 0.016 19.478 0.000

WX1 2.184 0.268 8.136 0.000

WY1 0.340 0.023 14.790 0.000

Residual Variances

X1 0.000 0.000 999.000 999.000

X2 0.000 0.000 999.000 999.000

X3 0.000 0.000 999.000 999.000

X4 0.000 0.000 999.000 999.000

Y1 0.000 0.000 999.000 999.000

Y2 0.000 0.000 999.000 999.000

Y3 0.000 0.000 999.000 999.000

Y4 0.000 0.000 999.000 999.000

WX2 1.420 0.078 18.234 0.000

WX3 1.519 0.089 17.128 0.000

WX4 1.399 0.080 17.433 0.000

WY2 0.204 0.010 20.850 0.000

WY3 0.080 0.013 6.042 0.000

WY4 0.054 0.020 2.693 0.007

QUALITY OF NUMERICAL RESULTS

Condition Number for the Information Matrix 0.500E-04

(ratio of smallest to largest eigenvalue)

STANDARDIZED MODEL RESULTS

STDYX Standardization

Two-Tailed

Estimate S.E. Est./S.E. P-Value

IX BY

X1 0.549 0.070 7.820 0.000

X2 0.533 0.068 7.880 0.000

X3 0.513 0.066 7.812 0.000

X4 0.500 0.064 7.778 0.000

IY BY

Y1 0.648 0.018 35.721 0.000

Y2 0.716 0.013 53.900 0.000

Y3 0.831 0.020 42.032 0.000

Y4 0.853 0.018 47.622 0.000

WX1 BY

X1 0.808 0.048 16.986 0.000

WX2 BY

X2 0.811 0.044 18.257 0.000

WX3 BY

X3 0.819 0.042 19.545 0.000

WX4 BY

X4 0.813 0.040 20.097 0.000

WY1 BY

Y1 0.675 0.021 32.195 0.000

WY2 BY

Y2 0.619 0.014 45.844 0.000

WY3 BY

Y3 0.431 0.036 12.102 0.000

WY4 BY

Y4 0.396 0.036 10.877 0.000

WX2 ON

WX1 0.573 0.055 10.320 0.000

WY1 0.117 0.034 3.452 0.001

WX3 ON

WX2 0.620 0.045 13.872 0.000

WY2 0.053 0.033 1.600 0.110

WX4 ON

WX3 0.679 0.038 18.052 0.000

WY3 0.027 0.042 0.641 0.521

WY2 ON

WY1 0.299 0.037 8.072 0.000

WX1 0.120 0.036 3.305 0.001

WY3 ON

WY2 0.125 0.051 2.469 0.014

WX2 0.138 0.068 2.028 0.043

WY4 ON

WY3 -0.363 0.231 -1.576 0.115

WX3 0.402 0.154 2.610 0.009

X1 ON

SEX 0.156 0.023 6.813 0.000

SES 0.149 0.023 6.604 0.000

X2 ON

SEX 0.161 0.022 7.411 0.000

SES 0.181 0.022 8.391 0.000

X3 ON

SEX 0.180 0.022 8.174 0.000

SES 0.188 0.022 8.708 0.000

X4 ON

SEX 0.226 0.023 9.825 0.000

SES 0.199 0.023 8.642 0.000

Y1 ON

SEX 0.060 0.021 2.871 0.004

SES 0.289 0.020 14.155 0.000

Y2 ON

SEX 0.036 0.020 1.816 0.069

SES 0.302 0.019 15.752 0.000

Y3 ON

SEX 0.005 0.020 0.235 0.814

SES 0.350 0.020 17.803 0.000

Y4 ON

SEX 0.030 0.020 1.476 0.140

SES 0.337 0.020 16.847 0.000

IX WITH

IY 0.742 0.074 10.071 0.000

WX1 WITH

WY1 0.361 0.033 10.962 0.000

WX2 WITH

WY2 0.179 0.033 5.456 0.000

WX3 WITH

WY3 0.266 0.060 4.461 0.000

WX4 WITH

WY4 0.217 0.087 2.503 0.012

IY WITH

WY1 0.043 0.047 0.927 0.354

Means

IX 0.000 0.000 999.000 999.000

IY 0.000 0.000 999.000 999.000

WX1 0.000 0.000 999.000 999.000

WY1 0.000 0.000 999.000 999.000

Intercepts

X1 2.269 0.093 24.302 0.000

X2 2.256 0.090 25.028 0.000

X3 1.979 0.086 23.067 0.000

X4 1.663 0.085 19.486 0.000

Y1 5.017 0.108 46.635 0.000

Y2 6.668 0.133 50.091 0.000

Y3 8.570 0.142 60.247 0.000

Y4 9.295 0.186 50.012 0.000

WX2 0.000 0.000 999.000 999.000

WX3 0.000 0.000 999.000 999.000

WX4 0.000 0.000 999.000 999.000

WY2 0.000 0.000 999.000 999.000

WY3 0.000 0.000 999.000 999.000

WY4 0.000 0.000 999.000 999.000

Variances

IX 1.000 0.000 999.000 999.000

IY 1.000 0.000 999.000 999.000

WX1 1.000 0.000 999.000 999.000

WY1 1.000 0.000 999.000 999.000

Residual Variances

X1 0.000 999.000 999.000 999.000

X2 0.000 999.000 999.000 999.000

X3 0.000 999.000 999.000 999.000

X4 0.000 999.000 999.000 999.000

Y1 0.000 999.000 999.000 999.000

Y2 0.000 999.000 999.000 999.000

Y3 0.000 999.000 999.000 999.000

Y4 0.000 999.000 999.000 999.000

WX2 0.610 0.060 10.182 0.000

WX3 0.593 0.055 10.804 0.000

WX4 0.527 0.053 10.017 0.000

WY2 0.870 0.028 31.620 0.000

WY3 0.955 0.026 36.146 0.000

WY4 0.800 0.169 4.737 0.000

R-SQUARE

Observed Two-Tailed

Variable Estimate S.E. Est./S.E. P-Value

X1 1.000 999.000 999.000 999.000

X2 1.000 999.000 999.000 999.000

X3 1.000 999.000 999.000 999.000

X4 1.000 999.000 999.000 999.000

Y1 1.000 999.000 999.000 999.000

Y2 1.000 999.000 999.000 999.000

Y3 1.000 999.000 999.000 999.000

Y4 1.000 999.000 999.000 999.000

Latent Two-Tailed

Variable Estimate S.E. Est./S.E. P-Value

WX2 0.390 0.060 6.512 0.000

WX3 0.407 0.055 7.413 0.000

WX4 0.473 0.053 9.008 0.000

WY2 0.130 0.028 4.711 0.000

WY3 0.045 0.026 1.703 0.088

WY4 0.200 0.169 1.188 0.235

RESIDUAL OUTPUT

ESTIMATED MODEL AND RESIDUALS (OBSERVED - ESTIMATED)

Model Estimated Means

X1 X2 X3 X4 Y1

________ ________ ________ ________ ________

5.011 5.162 4.934 4.707 4.492

Model Estimated Means

Y2 Y3 Y4 SEX SES

________ ________ ________ ________ ________

5.299 5.786 6.159 1.507 0.016

Residuals for Means

X1 X2 X3 X4 Y1

________ ________ ________ ________ ________

0.004 -0.003 0.000 0.001 0.001

Residuals for Means

Y2 Y3 Y4 SEX SES

________ ________ ________ ________ ________

-0.001 0.000 0.000 0.000 0.000

Standardized Residuals (z-scores) for Means

X1 X2 X3 X4 Y1

________ ________ ________ ________ ________

1.641 -0.957 0.178 999.000 999.000

Standardized Residuals (z-scores) for Means

Y2 Y3 Y4 SEX SES

________ ________ ________ ________ ________

-3.121 0.482 -1.067 0.000 0.000

Normalized Residuals for Means

X1 X2 X3 X4 Y1

________ ________ ________ ________ ________

0.101 -0.059 0.007 0.024 0.048

Normalized Residuals for Means

Y2 Y3 Y4 SEX SES

________ ________ ________ ________ ________

-0.035 0.021 -0.032 0.000 0.000

Model Estimated Covariances

X1 X2 X3 X4 Y1

________ ________ ________ ________ ________

X1 3.343

X2 2.569 3.539

X3 2.134 2.787 3.822

X4 1.891 2.361 3.101 4.022

Y1 0.809 0.808 0.732 0.683 0.746

Y2 0.651 0.730 0.701 0.656 0.487

Y3 0.529 0.579 0.654 0.628 0.401

Y4 0.526 0.577 0.626 0.654 0.392

SEX 0.140 0.149 0.173 0.223 0.024

SES 0.281 0.351 0.379 0.411 0.261

Model Estimated Covariances

Y2 Y3 Y4 SEX SES

________ ________ ________ ________ ________

Y2 0.611

Y3 0.396 0.454

Y4 0.374 0.348 0.430

SEX 0.012 0.000 0.008 0.250

SES 0.247 0.247 0.231 -0.008 1.098

Model Estimated Correlations

X1 X2 X3 X4 Y1

________ ________ ________ ________ ________

X1 1.000

X2 0.747 1.000

X3 0.597 0.758 1.000

X4 0.516 0.626 0.791 1.000

Y1 0.512 0.497 0.434 0.395 1.000

Y2 0.455 0.497 0.459 0.419 0.722

Y3 0.430 0.457 0.497 0.465 0.690

Y4 0.439 0.468 0.488 0.497 0.692

SEX 0.153 0.158 0.177 0.223 0.055

SES 0.147 0.178 0.185 0.196 0.288

Model Estimated Correlations

Y2 Y3 Y4 SEX SES

________ ________ ________ ________ ________

Y2 1.000

Y3 0.752 1.000

Y4 0.730 0.788 1.000

SEX 0.032 -0.001 0.025 1.000

SES 0.301 0.350 0.336 -0.016 1.000

Residuals for Covariances

X1 X2 X3 X4 Y1

________ ________ ________ ________ ________

X1 0.009

X2 0.000 -0.010

X3 0.064 0.016 0.042

X4 0.078 -0.105 0.006 -0.026

Y1 0.003 0.004 0.061 0.017 0.002

Y2 -0.004 -0.002 0.017 -0.045 0.000

Y3 0.013 0.004 0.022 -0.008 0.015

Y4 -0.009 -0.005 0.014 -0.016 -0.013

SEX 0.000 0.000 0.000 0.000 0.000

SES -0.002 0.001 0.001 0.001 0.000

Residuals for Covariances

Y2 Y3 Y4 SEX SES

________ ________ ________ ________ ________

Y2 -0.002

Y3 0.004 0.009

Y4 -0.006 0.001 -0.008

SEX 0.000 0.000 0.000 0.000

SES 0.000 0.000 -0.001 0.000 0.000

Residuals for Correlations

X1 X2 X3 X4 Y1

________ ________ ________ ________ ________

X1 0.000

X2 0.000 0.000

X3 0.014 0.001 0.000

X4 0.022 -0.025 0.000 0.000

Y1 0.000 0.002 0.033 0.011 0.000

Y2 -0.002 0.000 0.009 -0.027 0.000

Y3 0.006 -0.001 0.009 -0.009 0.019

Y4 -0.004 0.001 0.013 -0.006 -0.017

SEX 0.000 0.000 -0.001 0.001 0.001

SES -0.001 0.001 -0.001 0.001 -0.001

Residuals for Correlations

Y2 Y3 Y4 SEX SES

________ ________ ________ ________ ________

Y2 0.000

Y3 0.001 0.000

Y4 -0.004 0.001 0.000

SEX 0.000 0.000 0.001 0.000

SES 0.001 -0.003 0.002 0.000 0.000

Standardized Residuals (z-scores) for Covariances

X1 X2 X3 X4 Y1

________ ________ ________ ________ ________

X1 1.483

X2 -0.041 999.000

X3 1.384 3.838 999.000

X4 1.923 -2.166 0.911 999.000

Y1 0.337 0.392 2.293 0.453 0.825

Y2 -0.563 -0.181 2.544 -1.645 0.000

Y3 1.012 0.354 2.971 -0.651 3.056

Y4 -0.570 -0.332 2.156 -2.115 -1.943

SEX 0.174 0.192 -0.733 999.000 999.000

SES 999.000 999.000 999.000 999.000 999.000

Standardized Residuals (z-scores) for Covariances

Y2 Y3 Y4 SEX SES

________ ________ ________ ________ ________

Y2 -1.686

Y3 1.507 1.814

Y4 999.000 999.000 999.000

SEX 999.000 -0.554 999.000 0.000

SES 999.000 999.000 999.000 0.000 0.000

Normalized Residuals for Covariances

X1 X2 X3 X4 Y1

________ ________ ________ ________ ________

X1 0.091

X2 -0.004 -0.104

X3 0.634 0.168 0.425

X4 0.712 -1.004 0.057 -0.254

Y1 0.071 0.087 1.424 0.346 0.082

Y2 -0.102 -0.054 0.465 -1.089 0.000

Y3 0.426 0.127 0.706 -0.225 1.015

Y4 -0.267 -0.158 0.448 -0.494 -0.805

SEX 0.005 0.005 -0.016 0.001 0.031

SES -0.045 0.025 0.019 0.030 -0.022

Normalized Residuals for Covariances

Y2 Y3 Y4 SEX SES

________ ________ ________ ________ ________

Y2 -0.091

Y3 0.250 0.617

Y4 -0.391 0.066 -0.500

SEX 0.008 -0.020 0.017 0.000

SES 0.028 0.019 -0.040 0.000 0.000

Beginning Time: 15:13:14

Ending Time: 15:13:15

Elapsed Time: 00:00:01

MUTHEN & MUTHEN

3463 Stoner Ave.

Los Angeles, CA 90066

Tel: (310) 391-9971

Fax: (310) 391-8971

Web: www.StatModel.com

Support: Support@StatModel.com

Copyright (c) 1998-2021 Muthen & Muthen

B. Reading for fun and reading achievement of twin 1

Mplus VERSION 8.7

MUTHEN & MUTHEN

03/30/2023 3:13 PM

INPUT INSTRUCTIONS

TITLE: sex ses rE1-rE4 rF1-rF4 rN1-rN4 mE1-mE4 mP1-mP4 mG1-mG4 mN1-mN4

DATA: FILE IS 4.4Tw1noDssAllData.dat;

VARIABLE: NAMES ARE sex ses rE1-rE4 X1-X4 Y1-Y4 mE1-mE4 mP1-mP4 mG1-mG4 mN1-mN4;

USEVARIABLES ARE sex ses X1 X2 X3 X4 Y1 Y2 Y3 Y4 ;

MISSING ARE ALL (-999);

ANALYSIS: Estimator=MLR ;

ANALYSIS: MODEL=NOCOV; !Exog vars correlated by default, but this fixes ALL corrs=0. Use W

MODEL:

!___Intercept factor !___BY statements

ix BY x1@1 x2@1 x3@1 x4@1;

iy BY y1@1 y2@1 y3@1 y4@1;

!___Covariates

x1 x2 x3 x4 y1 y2 y3 y4 ON sex;

x1 x2 x3 x4 y1 y2 y3 y4 ON ses;

!___Latent variables

Wx1 BY x1@1;

Wx2 BY x2@1;

Wx3 BY x3@1;

Wx4 BY x4@1;

Wy1 BY y1@1;

Wy2 BY y2@1;

Wy3 BY y3@1;

Wy4 BY y4@1;

!___Autoregressions !___ON statements

Wx2 ON Wx1;

Wx3 ON Wx2;

Wx4 ON Wx3;

Wy2 ON Wy1;

Wy3 ON Wy2;

Wy4 ON Wy3;

!___Crosslagged paths

Wy2 ON Wx1;

Wy3 ON Wx2;

Wy4 ON Wx3;

Wx2 ON Wy1;

Wx3 ON Wy2;

Wx4 ON Wy3;

!__VARIANCE STRUCTURE:

!____Variables estimated/fixed

x1-x4@0;

y1-y4@0;

Wx1-Wx4;

Wy1-Wy4;

ix;

iy;

!____WITH statements

ix WITH iy;

Wx1 WITH Wy1;

Wx2 WITH Wy2;

Wx3 WITH Wy3;

Wx4 WITH Wy4;

!ix WITH Wx1;

iy WITH Wy1;

!__MEAN STRUCTURE: !____Variables estimated/fixed

!results identical if below 2 lines are omitted (as model seems to estimate manifest-me

! but here i explicitly estimate them (so is visible)!

[x1-x4];

[y1-y4];

!results same if below 2 lines are omitted, but adding in residual-means is no cost to

[Wx1-Wx4@0];

[Wy1-Wy4@0];

!do not estimate below means, model becomes unidentified despite indentical vars n cova

! Hence, means below are fixed at zero, or omit these 2 lines entirely and results will

[ix @0];

[iy @0];

OUTPUT: TECH1 TECH4 STDYX SAMPSTAT RESIDUAL;

*** WARNING

Input line exceeded 90 characters. Some input may be truncated.

ANALYSIS: MODEL=NOCOV; !Exog vars correlated by default, but this fixes ALL corrs=0. Use WI

*** WARNING

Input line exceeded 90 characters. Some input may be truncated.

!results identical if below 2 lines are omitted (as model seems to estimate manifest-mea

*** WARNING

Input line exceeded 90 characters. Some input may be truncated.

!results same if below 2 lines are omitted, but adding in residual-means is no cost to m

*** WARNING

Input line exceeded 90 characters. Some input may be truncated.

!do not estimate below means, model becomes unidentified despite indentical vars n covar

*** WARNING

Data set contains cases with missing on x-variables.

These cases were not included in the analysis.

Number of cases with missing on x-variables: 9

5 WARNING(S) FOUND IN THE INPUT INSTRUCTIONS

sex ses rE1-rE4 rF1-rF4 rN1-rN4 mE1-mE4 mP1-mP4 mG1-mG4 mN1-mN4

SUMMARY OF ANALYSIS

Number of groups 1

Number of observations 2695

Number of dependent variables 8

Number of independent variables 2

Number of continuous latent variables 10

Observed dependent variables

Continuous

X1 X2 X3 X4 Y1 Y2

Y3 Y4

Observed independent variables

SEX SES

Continuous latent variables

IX IY WX1 WX2 WX3 WX4

WY1 WY2 WY3 WY4

Estimator MLR

Information matrix OBSERVED

Maximum number of iterations 1000

Convergence criterion 0.500D-04

Maximum number of steepest descent iterations 20

Maximum number of iterations for H1 2000

Convergence criterion for H1 0.100D-03

Input data file(s)

4.4Tw1noDssAllData.dat

Input data format FREE

SUMMARY OF DATA

Number of missing data patterns 127

COVARIANCE COVERAGE OF DATA

Minimum covariance coverage value 0.100

PROPORTION OF DATA PRESENT

Covariance Coverage

X1 X2 X3 X4 Y1

________ ________ ________ ________ ________

X1 0.539

X2 0.443 0.571

X3 0.322 0.422 0.536

X4 0.203 0.300 0.383 0.469

Y1 0.506 0.452 0.338 0.226 0.650

Y2 0.447 0.529 0.429 0.318 0.568

Y3 0.347 0.437 0.492 0.391 0.448

Y4 0.231 0.327 0.392 0.425 0.310

SEX 0.539 0.571 0.536 0.469 0.650

SES 0.539 0.571 0.536 0.469 0.650

Covariance Coverage

Y2 Y3 Y4 SEX SES

________ ________ ________ ________ ________

Y2 0.718

Y3 0.580 0.696

Y4 0.439 0.535 0.607

SEX 0.718 0.696 0.607 1.000

SES 0.718 0.696 0.607 1.000 1.000

SAMPLE STATISTICS

ESTIMATED SAMPLE STATISTICS

Means

X1 X2 X3 X4 Y1

________ ________ ________ ________ ________

4.967 4.987 4.635 4.217 4.495

Means

Y2 Y3 Y4 SEX SES

________ ________ ________ ________ ________

5.301 5.786 6.158 1.507 0.016

Covariances

X1 X2 X3 X4 Y1

________ ________ ________ ________ ________

X1 3.007

X2 2.089 3.345

X3 1.745 2.413 3.909

X4 1.497 1.935 3.050 4.545

Y1 0.536 0.657 0.618 0.643 0.746

Y2 0.449 0.612 0.561 0.606 0.487

Y3 0.356 0.471 0.546 0.596 0.417

Y4 0.343 0.475 0.528 0.617 0.379

SEX 0.106 0.107 0.138 0.204 0.023

SES 0.305 0.430 0.463 0.484 0.260

Covariances

Y2 Y3 Y4 SEX SES

________ ________ ________ ________ ________

Y2 0.609

Y3 0.400 0.464

Y4 0.369 0.350 0.424

SEX 0.011 -0.001 0.008 0.250

SES 0.246 0.248 0.232 -0.008 1.098

Correlations

X1 X2 X3 X4 Y1

________ ________ ________ ________ ________

X1 1.000

X2 0.659 1.000

X3 0.509 0.667 1.000

X4 0.405 0.496 0.724 1.000

Y1 0.358 0.416 0.362 0.349 1.000

Y2 0.331 0.429 0.364 0.364 0.723

Y3 0.301 0.378 0.406 0.411 0.709

Y4 0.304 0.399 0.410 0.444 0.674

SEX 0.122 0.118 0.140 0.191 0.053

SES 0.168 0.224 0.223 0.217 0.287

Correlations

Y2 Y3 Y4 SEX SES

________ ________ ________ ________ ________

Y2 1.000

Y3 0.753 1.000

Y4 0.726 0.790 1.000

SEX 0.029 -0.003 0.025 1.000

SES 0.301 0.348 0.340 -0.016 1.000

MAXIMUM LOG-LIKELIHOOD VALUE FOR THE UNRESTRICTED (H1) MODEL IS -22006.395

UNIVARIATE SAMPLE STATISTICS

UNIVARIATE HIGHER-ORDER MOMENT DESCRIPTIVE STATISTICS

Variable/ Mean/ Skewness/ Minimum/ % with Percentiles

Sample Size Variance Kurtosis Maximum Min/Max 20%/60% 40%/80% Median

X1 5.056 -0.942 1.000 7.64% 4.000 5.000 5.000

1453.000 2.936 0.208 7.000 21.89% 6.000 7.000

X2 5.024 -0.857 1.000 8.45% 4.000 5.000 5.000

1539.000 3.335 -0.204 7.000 25.28% 6.000 7.000

X3 4.648 -0.586 1.000 12.94% 3.000 5.000 5.000

1445.000 3.965 -0.845 7.000 21.25% 5.000 7.000

X4 4.281 -0.305 1.000 18.10% 2.000 4.000 5.000

1265.000 4.473 -1.238 7.000 19.21% 5.000 6.000

Y1 4.506 0.005 1.932 0.06% 3.780 4.309 4.523

1752.000 0.766 -0.036 7.600 0.06% 4.740 5.230

Y2 5.277 0.027 2.657 0.05% 4.647 5.100 5.253

1934.000 0.608 0.455 8.420 0.05% 5.480 5.929

Y3 5.770 0.083 3.662 0.05% 5.184 5.590 5.750

1876.000 0.455 -0.032 8.626 0.05% 5.928 6.338

Y4 6.149 -0.058 4.094 0.06% 5.623 6.000 6.150

1635.000 0.411 0.331 9.075 0.06% 6.294 6.670

SEX 1.507 -0.029 1.000 49.28% 1.000 1.000 2.000

2695.000 0.250 -1.999 2.000 50.72% 2.000 2.000

SES 0.016 0.131 -2.823 0.04% -0.887 -0.358 -0.056

2695.000 1.098 -0.569 2.935 0.04% 0.248 0.987

THE MODEL ESTIMATION TERMINATED NORMALLY

MODEL FIT INFORMATION

Number of Free Parameters 52

Loglikelihood

H0 Value -16115.642

H0 Scaling Correction Factor 1.1017

for MLR

H1 Value -16100.900

H1 Scaling Correction Factor 1.0839

for MLR

Information Criteria

Akaike (AIC) 32335.283

Bayesian (BIC) 32642.039

Sample-Size Adjusted BIC 32476.819

(n* = (n + 2) / 24)

Chi-Square Test of Model Fit

Value 30.458*

Degrees of Freedom 8

P-Value 0.0002

Scaling Correction Factor 0.9680

for MLR

* The chi-square value for MLM, MLMV, MLR, ULSMV, WLSM and WLSMV cannot be used

for chi-square difference testing in the regular way. MLM, MLR and WLSM

chi-square difference testing is described on the Mplus website. MLMV, WLSMV,

and ULSMV difference testing is done using the DIFFTEST option.

RMSEA (Root Mean Square Error Of Approximation)

Estimate 0.032

90 Percent C.I. 0.021 0.045

Probability RMSEA <= .05 0.991

CFI/TLI

CFI 0.997

TLI 0.981

Chi-Square Test of Model Fit for the Baseline Model

Value 6506.881

Degrees of Freedom 44

P-Value 0.0000

SRMR (Standardized Root Mean Square Residual)

Value 0.021

MODEL RESULTS

Two-Tailed

Estimate S.E. Est./S.E. P-Value

IX BY

X1 1.000 0.000 999.000 999.000

X2 1.000 0.000 999.000 999.000

X3 1.000 0.000 999.000 999.000

X4 1.000 0.000 999.000 999.000

IY BY

Y1 1.000 0.000 999.000 999.000

Y2 1.000 0.000 999.000 999.000

Y3 1.000 0.000 999.000 999.000

Y4 1.000 0.000 999.000 999.000

WX1 BY

X1 1.000 0.000 999.000 999.000

WX2 BY

X2 1.000 0.000 999.000 999.000

WX3 BY

X3 1.000 0.000 999.000 999.000

WX4 BY

X4 1.000 0.000 999.000 999.000

WY1 BY

Y1 1.000 0.000 999.000 999.000

WY2 BY

Y2 1.000 0.000 999.000 999.000

WY3 BY

Y3 1.000 0.000 999.000 999.000

WY4 BY

Y4 1.000 0.000 999.000 999.000

WX2 ON

WX1 0.515 0.063 8.217 0.000

WY1 0.384 0.095 4.063 0.000

WX3 ON

WX2 0.588 0.060 9.760 0.000

WY2 0.071 0.129 0.549 0.583

WX4 ON

WX3 0.649 0.037 17.452 0.000

WY3 0.620 0.264 2.349 0.019

WY2 ON

WY1 0.253 0.030 8.453 0.000

WX1 0.025 0.012 2.025 0.043

WY3 ON

WY2 0.097 0.042 2.307 0.021

WX2 0.011 0.012 0.976 0.329

WY4 ON

WY3 -0.173 0.189 -0.917 0.359

WX3 0.042 0.017 2.422 0.015

X1 ON

SEX 0.435 0.084 5.200 0.000

SES 0.278 0.039 7.178 0.000

X2 ON

SEX 0.443 0.083 5.324 0.000

SES 0.394 0.040 9.973 0.000

X3 ON

SEX 0.564 0.091 6.184 0.000

SES 0.424 0.043 9.954 0.000

X4 ON

SEX 0.834 0.103 8.063 0.000

SES 0.447 0.047 9.449 0.000

Y1 ON

SEX 0.099 0.036 2.750 0.006

SES 0.238 0.018 13.595 0.000

Y2 ON

SEX 0.054 0.031 1.710 0.087

SES 0.225 0.015 15.031 0.000

Y3 ON

SEX 0.005 0.027 0.167 0.867

SES 0.227 0.013 17.058 0.000

Y4 ON

SEX 0.039 0.027 1.433 0.152

SES 0.213 0.013 16.420 0.000

IX WITH

IY 0.303 0.035 8.766 0.000

WX1 WITH

WY1 0.186 0.039 4.802 0.000

WX2 WITH

WY2 0.096 0.021 4.596 0.000

WX3 WITH

WY3 0.086 0.023 3.696 0.000

WX4 WITH

WY4 0.096 0.022 4.273 0.000

IY WITH

WY1 0.029 0.016 1.776 0.076

Means

IX 0.000 0.000 999.000 999.000

IY 0.000 0.000 999.000 999.000

WX1 0.000 0.000 999.000 999.000

WY1 0.000 0.000 999.000 999.000

Intercepts

X1 4.307 0.136 31.565 0.000

X2 4.314 0.138 31.316 0.000

X3 3.774 0.149 25.374 0.000

X4 2.954 0.166 17.831 0.000

Y1 4.339 0.058 74.885 0.000

Y2 5.216 0.050 104.249 0.000

Y3 5.776 0.044 132.253 0.000

Y4 6.098 0.044 139.597 0.000

WX2 0.000 0.000 999.000 999.000

WX3 0.000 0.000 999.000 999.000

WX4 0.000 0.000 999.000 999.000

WY2 0.000 0.000 999.000 999.000

WY3 0.000 0.000 999.000 999.000

WY4 0.000 0.000 999.000 999.000

Variances

IX 0.742 0.251 2.954 0.003

IY 0.305 0.018 17.134 0.000

WX1 2.179 0.275 7.923 0.000

WY1 0.324 0.022 14.525 0.000

Residual Variances

X1 0.000 0.000 999.000 999.000

X2 0.000 0.000 999.000 999.000

X3 0.000 0.000 999.000 999.000

X4 0.000 0.000 999.000 999.000

Y1 0.000 0.000 999.000 999.000

Y2 0.000 0.000 999.000 999.000

Y3 0.000 0.000 999.000 999.000

Y4 0.000 0.000 999.000 999.000

WX2 1.675 0.096 17.529 0.000

WX3 2.019 0.106 18.976 0.000

WX4 2.009 0.105 19.199 0.000

WY2 0.205 0.010 20.379 0.000

WY3 0.089 0.015 5.969 0.000

WY4 0.067 0.019 3.596 0.000

QUALITY OF NUMERICAL RESULTS

Condition Number for the Information Matrix 0.523E-04

(ratio of smallest to largest eigenvalue)

STANDARDIZED MODEL RESULTS

STDYX Standardization

Two-Tailed

Estimate S.E. Est./S.E. P-Value

IX BY

X1 0.493 0.084 5.886 0.000

X2 0.472 0.079 5.935 0.000

X3 0.438 0.075 5.809 0.000

X4 0.407 0.069 5.937 0.000

IY BY

Y1 0.637 0.020 31.567 0.000

Y2 0.710 0.016 45.281 0.000

Y3 0.819 0.023 35.404 0.000

Y4 0.844 0.020 41.787 0.000

WX1 BY

X1 0.845 0.049 17.081 0.000

WX2 BY

X2 0.844 0.045 18.895 0.000

WX3 BY

X3 0.859 0.039 21.776 0.000

WX4 BY

X4 0.865 0.033 26.204 0.000

WY1 BY

Y1 0.657 0.021 31.476 0.000

WY2 BY

Y2 0.616 0.013 45.688 0.000

WY3 BY

Y3 0.450 0.039 11.562 0.000

WY4 BY

Y4 0.413 0.039 10.626 0.000

WX2 ON

WX1 0.494 0.063 7.885 0.000

WY1 0.142 0.036 3.968 0.000

WX3 ON

WX2 0.536 0.056 9.588 0.000

WY2 0.020 0.037 0.548 0.583

WX4 ON

WX3 0.600 0.038 15.806 0.000

WY3 0.103 0.043 2.411 0.016

WY2 ON

WY1 0.301 0.036 8.390 0.000

WX1 0.078 0.039 2.009 0.045

WY3 ON

WY2 0.154 0.057 2.719 0.007

WX2 0.058 0.061 0.957 0.339

WY4 ON

WY3 -0.195 0.212 -0.917 0.359

WX3 0.262 0.120 2.193 0.028

X1 ON

SEX 0.124 0.024 5.250 0.000

SES 0.167 0.023 7.251 0.000

X2 ON

SEX 0.121 0.023 5.390 0.000

SES 0.226 0.022 10.143 0.000

X3 ON

SEX 0.143 0.023 6.257 0.000

SES 0.226 0.023 10.040 0.000

X4 ON

SEX 0.197 0.024 8.171 0.000

SES 0.222 0.023 9.516 0.000

Y1 ON

SEX 0.057 0.021 2.754 0.006

SES 0.288 0.020 14.055 0.000

Y2 ON

SEX 0.034 0.020 1.712 0.087

SES 0.303 0.019 15.698 0.000

Y3 ON

SEX 0.003 0.020 0.167 0.867

SES 0.352 0.020 17.879 0.000

Y4 ON

SEX 0.030 0.021 1.436 0.151

SES 0.341 0.020 17.083 0.000

IX WITH

IY 0.637 0.091 7.015 0.000

WX1 WITH

WY1 0.222 0.039 5.730 0.000

WX2 WITH

WY2 0.164 0.034 4.860 0.000

WX3 WITH

WY3 0.203 0.055 3.667 0.000

WX4 WITH

WY4 0.262 0.078 3.374 0.001

IY WITH

WY1 0.091 0.055 1.661 0.097

Means

IX 0.000 0.000 999.000 999.000

IY 0.000 0.000 999.000 999.000

WX1 0.000 0.000 999.000 999.000

WY1 0.000 0.000 999.000 999.000

Intercepts

X1 2.466 0.104 23.681 0.000

X2 2.363 0.096 24.619 0.000

X3 1.918 0.088 21.812 0.000

X4 1.397 0.085 16.513 0.000

Y1 5.011 0.108 46.551 0.000

Y2 6.710 0.133 50.334 0.000

Y3 8.567 0.142 60.395 0.000

Y4 9.326 0.185 50.482 0.000

WX2 0.000 0.000 999.000 999.000

WX3 0.000 0.000 999.000 999.000

WX4 0.000 0.000 999.000 999.000

WY2 0.000 0.000 999.000 999.000

WY3 0.000 0.000 999.000 999.000

WY4 0.000 0.000 999.000 999.000

Variances

IX 1.000 0.000 999.000 999.000

IY 1.000 0.000 999.000 999.000

WX1 1.000 0.000 999.000 999.000

WY1 1.000 0.000 999.000 999.000

Residual Variances

X1 0.000 999.000 999.000 999.000

X2 0.000 999.000 999.000 999.000

X3 0.000 999.000 999.000 999.000

X4 0.000 999.000 999.000 999.000

Y1 0.000 999.000 999.000 999.000

Y2 0.000 999.000 999.000 999.000

Y3 0.000 999.000 999.000 999.000

Y4 0.000 999.000 999.000 999.000

WX2 0.705 0.061 11.542 0.000

WX3 0.707 0.061 11.655 0.000

WX4 0.602 0.046 13.036 0.000

WY2 0.893 0.025 36.050 0.000

WY3 0.969 0.021 46.256 0.000

WY4 0.916 0.095 9.637 0.000

R-SQUARE

Observed Two-Tailed

Variable Estimate S.E. Est./S.E. P-Value

X1 1.000 999.000 999.000 999.000

X2 1.000 999.000 999.000 999.000

X3 1.000 999.000 999.000 999.000

X4 1.000 999.000 999.000 999.000

Y1 1.000 999.000 999.000 999.000

Y2 1.000 999.000 999.000 999.000

Y3 1.000 999.000 999.000 999.000

Y4 1.000 999.000 999.000 999.000

Latent Two-Tailed

Variable Estimate S.E. Est./S.E. P-Value

WX2 0.295 0.061 4.824 0.000

WX3 0.293 0.061 4.837 0.000

WX4 0.398 0.046 8.628 0.000

WY2 0.107 0.025 4.324 0.000

WY3 0.031 0.021 1.498 0.134

WY4 0.084 0.095 0.883 0.377

RESIDUAL OUTPUT

ESTIMATED MODEL AND RESIDUALS (OBSERVED - ESTIMATED)

Model Estimated Means

X1 X2 X3 X4 Y1

________ ________ ________ ________ ________

4.967 4.988 4.631 4.217 4.493

Model Estimated Means

Y2 Y3 Y4 SEX SES

________ ________ ________ ________ ________

5.300 5.786 6.160 1.507 0.016

Residuals for Means

X1 X2 X3 X4 Y1

________ ________ ________ ________ ________

0.000 -0.002 0.004 0.000 0.002

Residuals for Means

Y2 Y3 Y4 SEX SES

________ ________ ________ ________ ________

0.001 0.000 -0.002 0.000 0.000

Standardized Residuals (z-scores) for Means

X1 X2 X3 X4 Y1

________ ________ ________ ________ ________

999.000 -0.757 999.000 -0.612 2.779

Standardized Residuals (z-scores) for Means

Y2 Y3 Y4 SEX SES

________ ________ ________ ________ ________

0.951 -0.411 999.000 0.000 0.000

Normalized Residuals for Means

X1 X2 X3 X4 Y1

________ ________ ________ ________ ________

0.011 -0.035 0.087 -0.009 0.125

Normalized Residuals for Means

Y2 Y3 Y4 SEX SES

________ ________ ________ ________ ________

0.052 -0.021 -0.109 0.000 0.000

Model Estimated Covariances

X1 X2 X3 X4 Y1

________ ________ ________ ________ ________

X1 3.051

X2 2.102 3.334

X3 1.640 2.394 3.872

X4 1.441 1.966 2.987 4.468

Y1 0.572 0.647 0.569 0.538 0.750

Y2 0.479 0.598 0.544 0.523 0.487

Y3 0.395 0.456 0.529 0.550 0.404

Y4 0.397 0.461 0.513 0.572 0.393

SEX 0.106 0.108 0.137 0.205 0.023

SES 0.302 0.429 0.461 0.484 0.261

Model Estimated Covariances

Y2 Y3 Y4 SEX SES

________ ________ ________ ________ ________

Y2 0.604

Y3 0.393 0.454

Y4 0.366 0.347 0.428

SEX 0.012 -0.001 0.008 0.250

SES 0.246 0.249 0.233 -0.008 1.098

Model Estimated Correlations

X1 X2 X3 X4 Y1

________ ________ ________ ________ ________

X1 1.000

X2 0.659 1.000

X3 0.477 0.666 1.000

X4 0.390 0.509 0.718 1.000

Y1 0.378 0.409 0.334 0.294 1.000

Y2 0.352 0.421 0.356 0.318 0.723

Y3 0.336 0.371 0.399 0.386 0.692

Y4 0.347 0.386 0.399 0.414 0.695

SEX 0.122 0.118 0.140 0.194 0.053

SES 0.165 0.224 0.224 0.219 0.287

Model Estimated Correlations

Y2 Y3 Y4 SEX SES

________ ________ ________ ________ ________

Y2 1.000

Y3 0.750 1.000

Y4 0.720 0.788 1.000

SEX 0.030 -0.002 0.024 1.000

SES 0.303 0.352 0.340 -0.016 1.000

Residuals for Covariances

X1 X2 X3 X4 Y1

________ ________ ________ ________ ________

X1 -0.044

X2 -0.014 0.011

X3 0.106 0.018 0.037

X4 0.056 -0.030 0.063 0.077

Y1 -0.036 0.010 0.049 0.104 -0.004

Y2 -0.030 0.014 0.017 0.083 0.001

Y3 -0.040 0.015 0.017 0.047 0.013

Y4 -0.054 0.014 0.015 0.044 -0.014

SEX -0.001 0.000 0.001 -0.001 0.000

SES 0.003 0.001 0.002 0.000 -0.001

Residuals for Covariances

Y2 Y3 Y4 SEX SES

________ ________ ________ ________ ________

Y2 0.005

Y3 0.008 0.009

Y4 0.003 0.003 -0.004

SEX 0.000 0.000 0.000 0.000

SES 0.000 0.000 -0.001 0.000 0.000

Residuals for Correlations

X1 X2 X3 X4 Y1

________ ________ ________ ________ ________

X1 0.000

X2 -0.001 0.000

X3 0.032 0.001 0.000

X4 0.015 -0.013 0.006 0.000

Y1 -0.020 0.007 0.028 0.055 0.000

Y2 -0.021 0.007 0.008 0.046 0.000

Y3 -0.035 0.008 0.007 0.025 0.017

Y4 -0.043 0.013 0.011 0.030 -0.021

SEX 0.000 0.000 0.000 -0.002 0.000

SES 0.003 0.000 0.000 -0.002 0.000

Residuals for Correlations

Y2 Y3 Y4 SEX SES

________ ________ ________ ________ ________

Y2 0.000

Y3 0.003 0.000

Y4 0.007 0.002 0.000

SEX 0.000 0.000 0.001 0.000

SES -0.001 -0.004 0.000 0.000 0.000

Standardized Residuals (z-scores) for Covariances

X1 X2 X3 X4 Y1

________ ________ ________ ________ ________

X1 999.000

X2 999.000 0.597

X3 2.050 999.000 999.000

X4 3.429 -0.512 999.000 999.000

Y1 -5.831 999.000 1.595 2.440 999.000

Y2 -3.549 1.082 2.049 2.554 999.000

Y3 -2.758 1.348 1.480 2.832 2.672

Y4 -3.077 0.804 1.646 2.872 -2.280

SEX -0.543 -0.056 999.000 -0.755 0.612

SES 0.617 999.000 999.000 999.000 999.000

Standardized Residuals (z-scores) for Covariances

Y2 Y3 Y4 SEX SES

________ ________ ________ ________ ________

Y2 1.206

Y3 2.096 1.865

Y4 999.000 1.796 999.000

SEX -1.025 -0.626 999.000 0.000

SES 0.000 999.000 999.000 0.000 0.000

Normalized Residuals for Covariances

X1 X2 X3 X4 Y1

________ ________ ________ ________ ________

X1 -0.388

X2 -0.134 0.103

X3 1.015 0.176 0.344

X4 0.486 -0.272 0.567 0.704

Y1 -0.902 0.237 1.074 1.974 -0.166

Y2 -0.833 0.394 0.455 1.845 0.031

Y3 -1.279 0.476 0.511 1.237 0.869

Y4 -1.569 0.435 0.435 1.225 -0.907

SEX -0.026 -0.002 0.032 -0.030 0.009

SES 0.068 0.014 0.034 0.006 -0.028

Normalized Residuals for Covariances

Y2 Y3 Y4 SEX SES

________ ________ ________ ________ ________

Y2 0.238

Y3 0.530 0.648

Y4 0.229 0.240 -0.241

SEX -0.018 -0.021 0.028 0.000

SES 0.000 -0.021 -0.076 0.000 0.000

Beginning Time: 15:13:09

Ending Time: 15:13:09

Elapsed Time: 00:00:00

MUTHEN & MUTHEN

3463 Stoner Ave.

Los Angeles, CA 90066

Tel: (310) 391-9971

Fax: (310) 391-8971

Web: www.StatModel.com

Support: Support@StatModel.com

Copyright (c) 1998-2021 Muthen & Muthen

C. Reading enjoyment and reading achievement of twin 2

Mplus VERSION 8.7

MUTHEN & MUTHEN

02/10/2023 10:05 AM

INPUT INSTRUCTIONS

TITLE: sex ses rE1-rE4 rF1-rF4 rN1-rN4 mE1-mE4 mP1-mP4 mG1-mG4 mN1-mN4

DATA: FILE IS 4.4Tw2noDssAllData.dat;

VARIABLE: NAMES ARE sex ses X1-X4 rF1-rF4 Y1-Y4 mE1-mE4 mP1-mP4 mG1-mG4 mN1-mN4;

USEVARIABLES ARE sex ses X1 X2 X3 X4 Y1 Y2 Y3 Y4 ;

MISSING ARE ALL (-999);

ANALYSIS: Estimator=MLR ;

ANALYSIS: MODEL=NOCOV; !Exog vars correlated by default, but this fixes ALL corrs=0. Use W

MODEL:

!___Intercept factor !___BY statements

ix BY x1@1 x2@1 x3@1 x4@1;

iy BY y1@1 y2@1 y3@1 y4@1;

!___Covariates

x1 x2 x3 x4 y1 y2 y3 y4 ON sex;

x1 x2 x3 x4 y1 y2 y3 y4 ON ses;

!___Latent variables

Wx1 BY x1@1;

Wx2 BY x2@1;

Wx3 BY x3@1;

Wx4 BY x4@1;

Wy1 BY y1@1;

Wy2 BY y2@1;

Wy3 BY y3@1;

Wy4 BY y4@1;

!___Autoregressions !___ON statements

Wx2 ON Wx1;

Wx3 ON Wx2;

Wx4 ON Wx3;

Wy2 ON Wy1;

Wy3 ON Wy2;

Wy4 ON Wy3;

!___Crosslagged paths

Wy2 ON Wx1;

Wy3 ON Wx2;

Wy4 ON Wx3;

Wx2 ON Wy1;

Wx3 ON Wy2;

Wx4 ON Wy3;

!__VARIANCE STRUCTURE:

!____Variables estimated/fixed

x1-x4@0;

y1-y4@0;

Wx1-Wx4;

Wy1-Wy4;

ix;

iy;

!____WITH statements

ix WITH iy;

Wx1 WITH Wy1;

Wx2 WITH Wy2;

Wx3 WITH Wy3;

Wx4 WITH Wy4;

!ix WITH Wx1;

iy WITH Wy1;

!__MEAN STRUCTURE: !____Variables estimated/fixed

!results identical if below 2 lines are omitted (as model seems to estimate manifest-me

! but here i explicitly estimate them (so is visible)!

[x1-x4];

[y1-y4];

!results same if below 2 lines are omitted, but adding in residual-means is no cost to

[Wx1-Wx4@0];

[Wy1-Wy4@0];

!do not estimate below means, model becomes unidentified despite indentical vars n cova

! Hence, means below are fixed at zero, or omit these 2 lines entirely and results will

[ix @0];

[iy @0];

OUTPUT: TECH1 TECH4 STDYX SAMPSTAT RESIDUAL;

*** WARNING

Input line exceeded 90 characters. Some input may be truncated.

ANALYSIS: MODEL=NOCOV; !Exog vars correlated by default, but this fixes ALL corrs=0. Use WI

*** WARNING

Input line exceeded 90 characters. Some input may be truncated.

!results identical if below 2 lines are omitted (as model seems to estimate manifest-mea

*** WARNING

Input line exceeded 90 characters. Some input may be truncated.

!results same if below 2 lines are omitted, but adding in residual-means is no cost to m

*** WARNING

Input line exceeded 90 characters. Some input may be truncated.

!do not estimate below means, model becomes unidentified despite indentical vars n covar

*** WARNING

Data set contains cases with missing on all variables.

These cases were not included in the analysis.

Number of cases with missing on all variables: 2

*** WARNING

Data set contains cases with missing on x-variables.

These cases were not included in the analysis.

Number of cases with missing on x-variables: 8

6 WARNING(S) FOUND IN THE INPUT INSTRUCTIONS

sex ses rE1-rE4 rF1-rF4 rN1-rN4 mE1-mE4 mP1-mP4 mG1-mG4 mN1-mN4

SUMMARY OF ANALYSIS

Number of groups 1

Number of observations 2695

Number of dependent variables 8

Number of independent variables 2

Number of continuous latent variables 10

Observed dependent variables

Continuous

X1 X2 X3 X4 Y1 Y2

Y3 Y4

Observed independent variables

SEX SES

Continuous latent variables

IX IY WX1 WX2 WX3 WX4

WY1 WY2 WY3 WY4

Estimator MLR

Information matrix OBSERVED

Maximum number of iterations 1000

Convergence criterion 0.500D-04

Maximum number of steepest descent iterations 20

Maximum number of iterations for H1 2000

Convergence criterion for H1 0.100D-03

Input data file(s)

4.4Tw2noDssAllData.dat

Input data format FREE

SUMMARY OF DATA

Number of missing data patterns 123

COVARIANCE COVERAGE OF DATA

Minimum covariance coverage value 0.100

PROPORTION OF DATA PRESENT

Covariance Coverage

X1 X2 X3 X4 Y1

________ ________ ________ ________ ________

X1 0.539

X2 0.441 0.569

X3 0.321 0.422 0.535

X4 0.202 0.299 0.383 0.470

Y1 0.506 0.449 0.338 0.227 0.652

Y2 0.447 0.525 0.428 0.318 0.570

Y3 0.347 0.435 0.490 0.391 0.449

Y4 0.232 0.326 0.391 0.424 0.310

SEX 0.539 0.569 0.535 0.470 0.652

SES 0.539 0.569 0.535 0.470 0.652

Covariance Coverage

Y2 Y3 Y4 SEX SES

________ ________ ________ ________ ________

Y2 0.718

Y3 0.580 0.695

Y4 0.437 0.535 0.605

SEX 0.718 0.695 0.605 1.000

SES 0.718 0.695 0.605 1.000 1.000

SAMPLE STATISTICS

ESTIMATED SAMPLE STATISTICS

Means

X1 X2 X3 X4 Y1

________ ________ ________ ________ ________

5.070 5.234 5.013 4.788 4.506

Means

Y2 Y3 Y4 SEX SES

________ ________ ________ ________ ________

5.298 5.786 6.192 1.518 0.019

Covariances

X1 X2 X3 X4 Y1

________ ________ ________ ________ ________

X1 3.286

X2 2.403 3.268

X3 2.118 2.703 3.854

X4 1.956 2.164 3.013 4.002

Y1 0.783 0.832 0.789 0.677 0.767

Y2 0.665 0.736 0.783 0.685 0.532

Y3 0.562 0.609 0.728 0.660 0.439

Y4 0.537 0.579 0.709 0.666 0.414

SEX 0.134 0.120 0.135 0.222 0.018

SES 0.241 0.304 0.357 0.410 0.296

Covariances

Y2 Y3 Y4 SEX SES

________ ________ ________ ________ ________

Y2 0.639

Y3 0.408 0.473

Y4 0.399 0.366 0.454

SEX 0.023 0.007 0.010 0.250

SES 0.288 0.249 0.269 -0.006 1.107

Correlations

X1 X2 X3 X4 Y1

________ ________ ________ ________ ________

X1 1.000

X2 0.733 1.000

X3 0.595 0.762 1.000

X4 0.539 0.598 0.767 1.000

Y1 0.493 0.525 0.459 0.386 1.000

Y2 0.459 0.510 0.499 0.428 0.760

Y3 0.451 0.489 0.539 0.479 0.728

Y4 0.439 0.475 0.535 0.494 0.701

SEX 0.147 0.133 0.137 0.222 0.041

SES 0.126 0.160 0.173 0.195 0.321

Correlations

Y2 Y3 Y4 SEX SES

________ ________ ________ ________ ________

Y2 1.000

Y3 0.741 1.000

Y4 0.741 0.790 1.000

SEX 0.058 0.021 0.029 1.000

SES 0.342 0.343 0.379 -0.011 1.000

MAXIMUM LOG-LIKELIHOOD VALUE FOR THE UNRESTRICTED (H1) MODEL IS -21435.220

UNIVARIATE SAMPLE STATISTICS

UNIVARIATE HIGHER-ORDER MOMENT DESCRIPTIVE STATISTICS

Variable/ Mean/ Skewness/ Minimum/ % with Percentiles

Sample Size Variance Kurtosis Maximum Min/Max 20%/60% 40%/80% Median

X1 5.171 -0.669 1.000 3.58% 4.000 5.000 6.000

1452.000 3.153 -0.630 7.000 33.06% 6.000 7.000

X2 5.271 -0.783 1.000 4.31% 4.000 5.000 6.000

1533.000 3.256 -0.474 7.000 37.51% 6.000 7.000

X3 5.015 -0.613 1.000 7.21% 3.000 5.000 6.000

1443.000 3.964 -0.910 7.000 35.97% 6.000 7.000

X4 4.810 -0.472 1.000 8.61% 3.000 4.000 5.000

1266.000 4.075 -1.055 7.000 31.44% 6.000 7.000

Y1 4.516 0.026 1.810 0.06% 3.770 4.310 4.530

1757.000 0.754 -0.105 7.593 0.06% 4.740 5.240

Y2 5.277 0.112 2.620 0.10% 4.640 5.090 5.278

1935.000 0.630 0.255 8.044 0.05% 5.469 5.929

Y3 5.759 0.094 3.220 0.05% 5.170 5.589 5.766

1872.000 0.462 0.004 8.212 0.05% 5.928 6.310

Y4 6.176 -0.006 3.555 0.06% 5.614 6.000 6.191

1631.000 0.452 0.436 8.906 0.06% 6.347 6.730

SEX 1.518 -0.063 1.000 48.24% 1.000 1.000 2.000

2695.000 0.250 -1.972 3.000 0.04% 2.000 2.000

SES 0.019 0.164 -2.823 0.04% -0.886 -0.358 -0.055

2695.000 1.107 -0.427 5.000 0.04% 0.250 0.989

THE MODEL ESTIMATION TERMINATED NORMALLY

MODEL FIT INFORMATION

Number of Free Parameters 52

Loglikelihood

H0 Value -15523.702

H0 Scaling Correction Factor 1.1009

for MLR

H1 Value -15515.584

H1 Scaling Correction Factor 1.0904

for MLR

Information Criteria

Akaike (AIC) 31151.404

Bayesian (BIC) 31458.160

Sample-Size Adjusted BIC 31292.940

(n* = (n + 2) / 24)

Chi-Square Test of Model Fit

Value 15.893*

Degrees of Freedom 8

P-Value 0.0439

Scaling Correction Factor 1.0217

for MLR

* The chi-square value for MLM, MLMV, MLR, ULSMV, WLSM and WLSMV cannot be used

for chi-square difference testing in the regular way. MLM, MLR and WLSM

chi-square difference testing is described on the Mplus website. MLMV, WLSMV,

and ULSMV difference testing is done using the DIFFTEST option.

RMSEA (Root Mean Square Error Of Approximation)

Estimate 0.019

90 Percent C.I. 0.003 0.033

Probability RMSEA <= .05 1.000

CFI/TLI

CFI 0.999

TLI 0.994

Chi-Square Test of Model Fit for the Baseline Model

Value 7520.386

Degrees of Freedom 44

P-Value 0.0000

SRMR (Standardized Root Mean Square Residual)

Value 0.015

MODEL RESULTS

Two-Tailed

Estimate S.E. Est./S.E. P-Value

IX BY

X1 1.000 0.000 999.000 999.000

X2 1.000 0.000 999.000 999.000

X3 1.000 0.000 999.000 999.000

X4 1.000 0.000 999.000 999.000

IY BY

Y1 1.000 0.000 999.000 999.000

Y2 1.000 0.000 999.000 999.000

Y3 1.000 0.000 999.000 999.000

Y4 1.000 0.000 999.000 999.000

WX1 BY

X1 1.000 0.000 999.000 999.000

WX2 BY

X2 1.000 0.000 999.000 999.000

WX3 BY

X3 1.000 0.000 999.000 999.000

WX4 BY

X4 1.000 0.000 999.000 999.000

WY1 BY

Y1 1.000 0.000 999.000 999.000

WY2 BY

Y2 1.000 0.000 999.000 999.000

WY3 BY

Y3 1.000 0.000 999.000 999.000

WY4 BY

Y4 1.000 0.000 999.000 999.000

WX2 ON

WX1 0.476 0.061 7.861 0.000

WY1 0.620 0.081 7.678 0.000

WX3 ON

WX2 0.643 0.045 14.123 0.000

WY2 0.441 0.115 3.847 0.000

WX4 ON

WX3 0.627 0.043 14.530 0.000

WY3 0.159 0.208 0.764 0.445

WY2 ON

WY1 0.310 0.032 9.563 0.000

WX1 0.046 0.013 3.449 0.001

WY3 ON

WY2 0.047 0.034 1.402 0.161

WX2 0.064 0.013 4.822 0.000

WY4 ON

WY3 -0.239 0.148 -1.617 0.106

WX3 0.106 0.022 4.813 0.000

X1 ON

SEX 0.542 0.085 6.397 0.000

SES 0.223 0.041 5.502 0.000

X2 ON

SEX 0.484 0.081 5.989 0.000

SES 0.276 0.039 7.101 0.000

X3 ON

SEX 0.546 0.089 6.161 0.000

SES 0.326 0.042 7.797 0.000

X4 ON

SEX 0.893 0.094 9.467 0.000

SES 0.373 0.043 8.594 0.000

Y1 ON

SEX 0.078 0.036 2.166 0.030

SES 0.269 0.018 15.028 0.000

Y2 ON

SEX 0.098 0.032 3.097 0.002

SES 0.260 0.016 16.715 0.000

Y3 ON

SEX 0.035 0.028 1.226 0.220

SES 0.224 0.015 15.222 0.000

Y4 ON

SEX 0.045 0.027 1.654 0.098

SES 0.243 0.013 18.376 0.000

IX WITH

IY 0.391 0.040 9.811 0.000

WX1 WITH

WY1 0.298 0.042 7.116 0.000

WX2 WITH

WY2 0.095 0.018 5.194 0.000

WX3 WITH

WY3 0.129 0.021 6.290 0.000

WX4 WITH

WY4 0.033 0.020 1.651 0.099

IY WITH

WY1 0.026 0.012 2.124 0.034

Means

IX 0.000 0.000 999.000 999.000

IY 0.000 0.000 999.000 999.000

WX1 0.000 0.000 999.000 999.000

WY1 0.000 0.000 999.000 999.000

Intercepts

X1 4.238 0.139 30.542 0.000

X2 4.495 0.133 33.778 0.000

X3 4.178 0.144 28.943 0.000

X4 3.428 0.154 22.239 0.000

Y1 4.382 0.058 75.975 0.000

Y2 5.144 0.051 101.112 0.000

Y3 5.729 0.045 126.830 0.000

Y4 6.119 0.044 138.268 0.000

WX2 0.000 0.000 999.000 999.000

WX3 0.000 0.000 999.000 999.000

WX4 0.000 0.000 999.000 999.000

WY2 0.000 0.000 999.000 999.000

WY3 0.000 0.000 999.000 999.000

WY4 0.000 0.000 999.000 999.000

Variances

IX 1.145 0.218 5.253 0.000

IY 0.304 0.015 19.745 0.000

WX1 1.976 0.225 8.770 0.000

WY1 0.327 0.021 15.482 0.000

Residual Variances

X1 0.000 0.000 999.000 999.000

X2 0.000 0.000 999.000 999.000

X3 0.000 0.000 999.000 999.000

X4 0.000 0.000 999.000 999.000

Y1 0.000 0.000 999.000 999.000

Y2 0.000 0.000 999.000 999.000

Y3 0.000 0.000 999.000 999.000

Y4 0.000 0.000 999.000 999.000

WX2 1.272 0.077 16.470 0.000

WX3 1.486 0.087 17.176 0.000

WX4 1.473 0.078 18.838 0.000

WY2 0.201 0.010 20.002 0.000

WY3 0.094 0.013 7.258 0.000

WY4 0.068 0.015 4.488 0.000

QUALITY OF NUMERICAL RESULTS

Condition Number for the Information Matrix 0.662E-04

(ratio of smallest to largest eigenvalue)

STANDARDIZED MODEL RESULTS

STDYX Standardization

Two-Tailed

Estimate S.E. Est./S.E. P-Value

IX BY

X1 0.594 0.056 10.597 0.000

X2 0.588 0.055 10.641 0.000

X3 0.546 0.052 10.545 0.000

X4 0.535 0.052 10.374 0.000

IY BY

Y1 0.631 0.017 37.309 0.000

Y2 0.688 0.015 47.055 0.000

Y3 0.806 0.020 40.558 0.000

Y4 0.811 0.018 44.895 0.000

WX1 BY

X1 0.780 0.043 18.198 0.000

WX2 BY

X2 0.782 0.042 18.657 0.000

WX3 BY

X3 0.808 0.035 22.968 0.000

WX4 BY

X4 0.792 0.035 22.365 0.000

WY1 BY

Y1 0.655 0.019 34.287 0.000

WY2 BY

Y2 0.618 0.015 40.600 0.000

WY3 BY

Y3 0.473 0.032 14.725 0.000

WY4 BY

Y4 0.441 0.029 15.180 0.000

WX2 ON

WX1 0.471 0.060 7.862 0.000

WY1 0.249 0.037 6.688 0.000

WX3 ON

WX2 0.576 0.044 13.000 0.000

WY2 0.138 0.036 3.789 0.000

WX4 ON

WX3 0.627 0.040 15.609 0.000

WY3 0.032 0.042 0.763 0.445

WY2 ON

WY1 0.358 0.037 9.737 0.000

WX1 0.131 0.039 3.316 0.001

WY3 ON

WY2 0.073 0.049 1.473 0.141

WX2 0.283 0.060 4.696 0.000

WY4 ON

WY3 -0.257 0.158 -1.634 0.102

WX3 0.559 0.127 4.409 0.000

X1 ON

SEX 0.150 0.023 6.462 0.000

SES 0.130 0.024 5.528 0.000

X2 ON

SEX 0.133 0.022 6.054 0.000

SES 0.160 0.023 7.099 0.000

X3 ON

SEX 0.139 0.022 6.203 0.000

SES 0.175 0.022 7.858 0.000

X4 ON

SEX 0.223 0.023 9.650 0.000

SES 0.196 0.023 8.648 0.000

Y1 ON

SEX 0.045 0.021 2.169 0.030

SES 0.324 0.021 15.717 0.000

Y2 ON

SEX 0.061 0.020 3.125 0.002

SES 0.342 0.019 17.735 0.000

Y3 ON

SEX 0.025 0.021 1.227 0.220

SES 0.345 0.022 15.693 0.000

Y4 ON

SEX 0.033 0.020 1.659 0.097

SES 0.376 0.019 20.240 0.000

IX WITH

IY 0.664 0.043 15.507 0.000

WX1 WITH

WY1 0.371 0.036 10.326 0.000

WX2 WITH

WY2 0.187 0.034 5.464 0.000

WX3 WITH

WY3 0.345 0.053 6.514 0.000

WX4 WITH

WY4 0.106 0.064 1.649 0.099

IY WITH

WY1 0.081 0.040 2.011 0.044

Means

IX 0.000 0.000 999.000 999.000

IY 0.000 0.000 999.000 999.000

WX1 0.000 0.000 999.000 999.000

WY1 0.000 0.000 999.000 999.000

Intercepts

X1 2.352 0.094 25.083 0.000

X2 2.472 0.092 26.776 0.000

X3 2.130 0.086 24.903 0.000

X4 1.713 0.087 19.659 0.000

Y1 5.018 0.106 47.237 0.000

Y2 6.420 0.133 48.257 0.000

Y3 8.386 0.145 57.852 0.000

Y4 9.010 0.180 50.008 0.000

WX2 0.000 0.000 999.000 999.000

WX3 0.000 0.000 999.000 999.000

WX4 0.000 0.000 999.000 999.000

WY2 0.000 0.000 999.000 999.000

WY3 0.000 0.000 999.000 999.000

WY4 0.000 0.000 999.000 999.000

Variances

IX 1.000 0.000 999.000 999.000

IY 1.000 0.000 999.000 999.000

WX1 1.000 0.000 999.000 999.000

WY1 1.000 0.000 999.000 999.000

Residual Variances

X1 0.000 999.000 999.000 999.000

X2 0.000 999.000 999.000 999.000

X3 0.000 999.000 999.000 999.000

X4 0.000 999.000 999.000 999.000

Y1 0.000 999.000 999.000 999.000

Y2 0.000 999.000 999.000 999.000

Y3 0.000 999.000 999.000 999.000

Y4 0.000 999.000 999.000 999.000

WX2 0.629 0.054 11.641 0.000

WX3 0.592 0.053 11.089 0.000

WX4 0.587 0.048 12.161 0.000

WY2 0.820 0.031 26.480 0.000

WY3 0.900 0.039 23.222 0.000

WY4 0.752 0.099 7.567 0.000

R-SQUARE

Observed Two-Tailed

Variable Estimate S.E. Est./S.E. P-Value

X1 1.000 999.000 999.000 999.000

X2 1.000 999.000 999.000 999.000

X3 1.000 999.000 999.000 999.000

X4 1.000 999.000 999.000 999.000

Y1 1.000 999.000 999.000 999.000

Y2 1.000 999.000 999.000 999.000

Y3 1.000 999.000 999.000 999.000

Y4 1.000 999.000 999.000 999.000

Latent Two-Tailed

Variable Estimate S.E. Est./S.E. P-Value

WX2 0.371 0.054 6.857 0.000

WX3 0.408 0.053 7.653 0.000

WX4 0.413 0.048 8.564 0.000

WY2 0.180 0.031 5.805 0.000

WY3 0.100 0.039 2.583 0.010

WY4 0.248 0.099 2.494 0.013

RESIDUAL OUTPUT

ESTIMATED MODEL AND RESIDUALS (OBSERVED - ESTIMATED)

Model Estimated Means

X1 X2 X3 X4 Y1

________ ________ ________ ________ ________

5.065 5.236 5.014 4.790 4.506

Model Estimated Means

Y2 Y3 Y4 SEX SES

________ ________ ________ ________ ________

5.298 5.786 6.192 1.518 0.019

Residuals for Means

X1 X2 X3 X4 Y1

________ ________ ________ ________ ________

0.005 -0.002 -0.001 -0.003 0.001

Residuals for Means

Y2 Y3 Y4 SEX SES

________ ________ ________ ________ ________

0.000 0.000 0.000 0.000 0.000

Standardized Residuals (z-scores) for Means

X1 X2 X3 X4 Y1

________ ________ ________ ________ ________

999.000 -1.346 999.000 -1.038 0.442

Standardized Residuals (z-scores) for Means

Y2 Y3 Y4 SEX SES

________ ________ ________ ________ ________

-0.333 2.034 -0.306 0.000 0.000

Normalized Residuals for Means

X1 X2 X3 X4 Y1

________ ________ ________ ________ ________

0.125 -0.045 -0.021 -0.055 0.036

Normalized Residuals for Means

Y2 Y3 Y4 SEX SES

________ ________ ________ ________ ________

-0.014 0.029 -0.027 0.000 0.000

Model Estimated Covariances

X1 X2 X3 X4 Y1

________ ________ ________ ________ ________

X1 3.248

X2 2.403 3.308

X3 2.102 2.720 3.847

X4 1.873 2.272 3.012 4.006

Y1 0.765 0.842 0.784 0.702 0.763

Y2 0.651 0.751 0.782 0.702 0.531

Y3 0.532 0.621 0.722 0.662 0.425

Y4 0.522 0.601 0.708 0.674 0.425

SEX 0.134 0.120 0.135 0.221 0.018

SES 0.244 0.303 0.357 0.408 0.297

Model Estimated Covariances

Y2 Y3 Y4 SEX SES

________ ________ ________ ________ ________

Y2 0.642

Y3 0.406 0.467

Y4 0.405 0.366 0.461

SEX 0.023 0.007 0.010 0.250

SES 0.288 0.248 0.269 -0.006 1.107

Model Estimated Correlations

X1 X2 X3 X4 Y1

________ ________ ________ ________ ________

X1 1.000

X2 0.733 1.000

X3 0.595 0.762 1.000

X4 0.519 0.624 0.767 1.000

Y1 0.486 0.530 0.457 0.402 1.000

Y2 0.451 0.515 0.498 0.438 0.759

Y3 0.432 0.500 0.539 0.484 0.713

Y4 0.427 0.487 0.531 0.496 0.717

SEX 0.149 0.131 0.137 0.221 0.041

SES 0.129 0.158 0.173 0.194 0.323

Model Estimated Correlations

Y2 Y3 Y4 SEX SES

________ ________ ________ ________ ________

Y2 1.000

Y3 0.742 1.000

Y4 0.745 0.789 1.000

SEX 0.057 0.022 0.029 1.000

SES 0.341 0.345 0.376 -0.011 1.000

Residuals for Covariances

X1 X2 X3 X4 Y1

________ ________ ________ ________ ________

X1 0.038

X2 0.000 -0.040

X3 0.016 -0.017 0.007

X4 0.082 -0.108 0.001 -0.004

Y1 0.018 -0.011 0.006 -0.025 0.005

Y2 0.014 -0.015 0.001 -0.018 0.001

Y3 0.030 -0.013 0.006 -0.002 0.013

Y4 0.015 -0.022 0.001 -0.008 -0.011

SEX -0.001 0.001 0.000 0.001 0.000

SES -0.003 0.001 0.000 0.002 -0.001

Residuals for Covariances

Y2 Y3 Y4 SEX SES

________ ________ ________ ________ ________

Y2 -0.003

Y3 0.002 0.007

Y4 -0.006 0.000 -0.007

SEX 0.000 0.000 0.000 0.000

SES 0.000 0.001 0.000 0.000 0.000

Residuals for Correlations

X1 X2 X3 X4 Y1

________ ________ ________ ________ ________

X1 0.000

X2 0.000 0.000

X3 0.001 -0.001 0.000

X4 0.020 -0.026 0.000 0.000

Y1 0.007 -0.005 0.002 -0.015 0.000

Y2 0.008 -0.006 0.002 -0.010 0.001

Y3 0.019 -0.011 0.000 -0.005 0.015

Y4 0.013 -0.012 0.004 -0.002 -0.016

SEX -0.002 0.002 0.000 0.001 0.000

SES -0.002 0.001 0.000 0.001 -0.002

Residuals for Correlations

Y2 Y3 Y4 SEX SES

________ ________ ________ ________ ________

Y2 0.000

Y3 0.000 0.000

Y4 -0.004 0.000 0.000

SEX 0.000 0.000 0.000 0.000

SES 0.001 -0.002 0.003 0.000 0.000

Standardized Residuals (z-scores) for Covariances

X1 X2 X3 X4 Y1

________ ________ ________ ________ ________

X1 1.494

X2 -0.015 999.000

X3 0.328 999.000 999.000

X4 1.848 -2.353 999.000 999.000

Y1 1.593 -2.053 0.219 -0.710 1.061

Y2 1.815 -2.670 0.204 -0.660 999.000

Y3 1.707 -1.367 0.649 -0.189 2.091

Y4 0.941 -1.855 0.143 -0.912 -2.177

SEX 999.000 999.000 999.000 999.000 999.000

SES 999.000 999.000 999.000 999.000 999.000

Standardized Residuals (z-scores) for Covariances

Y2 Y3 Y4 SEX SES

________ ________ ________ ________ ________

Y2 999.000

Y3 999.000 1.297

Y4 -2.659 999.000 999.000

SEX 999.000 999.000 -0.270 0.000

SES 999.000 999.000 999.000 0.000 0.000

Normalized Residuals for Covariances

X1 X2 X3 X4 Y1

________ ________ ________ ________ ________

X1 0.384

X2 -0.001 -0.410

X3 0.164 -0.178 0.070

X4 0.764 -1.077 0.011 -0.034

Y1 0.446 -0.275 0.134 -0.522 0.193

Y2 0.391 -0.423 0.037 -0.423 0.042

Y3 0.937 -0.418 0.173 -0.065 0.846

Y4 0.454 -0.732 0.029 -0.251 -0.720

SEX -0.036 0.035 -0.007 0.040 -0.009

SES -0.069 0.023 -0.011 0.045 -0.034

Normalized Residuals for Covariances

Y2 Y3 Y4 SEX SES

________ ________ ________ ________ ________

Y2 -0.149

Y3 0.117 0.443

Y4 -0.387 0.004 -0.414

SEX 0.003 -0.003 -0.015 0.000

SES 0.006 0.034 0.022 0.000 0.000

Beginning Time: 10:05:01

Ending Time: 10:05:01

Elapsed Time: 00:00:00

MUTHEN & MUTHEN

3463 Stoner Ave.

Los Angeles, CA 90066

Tel: (310) 391-9971

Fax: (310) 391-8971

Web: www.StatModel.com

Support: Support@StatModel.com

Copyright (c) 1998-2021 Muthen & Muthen

D. Reading for fun and reading achievement of twin 2.

Mplus VERSION 8.7

MUTHEN & MUTHEN

02/10/2023 9:57 AM

INPUT INSTRUCTIONS

TITLE: sex ses rE1-rE4 rF1-rF4 rN1-rN4 mE1-mE4 mP1-mP4 mG1-mG4 mN1-mN4

DATA: FILE IS 4.4Tw2noDssAllData.dat;

VARIABLE: NAMES ARE sex ses rE1-rE4 X1-X4 Y1-Y4 mE1-mE4 mP1-mP4 mG1-mG4 mN1-mN4;

USEVARIABLES ARE sex ses X1 X2 X3 X4 Y1 Y2 Y3 Y4 ;

MISSING ARE ALL (-999);

ANALYSIS: Estimator=MLR ;

ANALYSIS: MODEL=NOCOV; !Exog vars correlated by default, but this fixes ALL corrs=0. Use W

MODEL:

!___Intercept factor !___BY statements

ix BY x1@1 x2@1 x3@1 x4@1;

iy BY y1@1 y2@1 y3@1 y4@1;

!___Covariates

x1 x2 x3 x4 y1 y2 y3 y4 ON sex;

x1 x2 x3 x4 y1 y2 y3 y4 ON ses;

!___Latent variables

Wx1 BY x1@1;

Wx2 BY x2@1;

Wx3 BY x3@1;

Wx4 BY x4@1;

Wy1 BY y1@1;

Wy2 BY y2@1;

Wy3 BY y3@1;

Wy4 BY y4@1;

!___Autoregressions !___ON statements

Wx2 ON Wx1;

Wx3 ON Wx2;

Wx4 ON Wx3;

Wy2 ON Wy1;

Wy3 ON Wy2;

Wy4 ON Wy3;

!___Crosslagged paths

Wy2 ON Wx1;

Wy3 ON Wx2;

Wy4 ON Wx3;

Wx2 ON Wy1;

Wx3 ON Wy2;

Wx4 ON Wy3;

!__VARIANCE STRUCTURE:

!____Variables estimated/fixed

x1-x4@0;

y1-y4@0;

Wx1-Wx4;

Wy1-Wy4;

ix;

iy;

!____WITH statements

ix WITH iy;

Wx1 WITH Wy1;

Wx2 WITH Wy2;

Wx3 WITH Wy3;

Wx4 WITH Wy4;

!ix WITH Wx1;

iy WITH Wy1;

!__MEAN STRUCTURE: !____Variables estimated/fixed

!results identical if below 2 lines are omitted (as model seems to estimate manifest-me

! but here i explicitly estimate them (so is visible)!

[x1-x4];

[y1-y4];

!results same if below 2 lines are omitted, but adding in residual-means is no cost to

[Wx1-Wx4@0];

[Wy1-Wy4@0];

!do not estimate below means, model becomes unidentified despite indentical vars n cova

! Hence, means below are fixed at zero, or omit these 2 lines entirely and results will

[ix @0];

[iy @0];

OUTPUT: TECH1 TECH4 STDYX SAMPSTAT RESIDUAL;

*** WARNING

Input line exceeded 90 characters. Some input may be truncated.

ANALYSIS: MODEL=NOCOV; !Exog vars correlated by default, but this fixes ALL corrs=0. Use WI

*** WARNING

Input line exceeded 90 characters. Some input may be truncated.

!results identical if below 2 lines are omitted (as model seems to estimate manifest-mea

*** WARNING

Input line exceeded 90 characters. Some input may be truncated.

!results same if below 2 lines are omitted, but adding in residual-means is no cost to m

*** WARNING

Input line exceeded 90 characters. Some input may be truncated.

!do not estimate below means, model becomes unidentified despite indentical vars n covar

*** WARNING

Data set contains cases with missing on x-variables.

These cases were not included in the analysis.

Number of cases with missing on x-variables: 10

5 WARNING(S) FOUND IN THE INPUT INSTRUCTIONS

sex ses rE1-rE4 rF1-rF4 rN1-rN4 mE1-mE4 mP1-mP4 mG1-mG4 mN1-mN4

SUMMARY OF ANALYSIS

Number of groups 1

Number of observations 2695

Number of dependent variables 8

Number of independent variables 2

Number of continuous latent variables 10

Observed dependent variables

Continuous

X1 X2 X3 X4 Y1 Y2

Y3 Y4

Observed independent variables

SEX SES

Continuous latent variables

IX IY WX1 WX2 WX3 WX4

WY1 WY2 WY3 WY4

Estimator MLR

Information matrix OBSERVED

Maximum number of iterations 1000

Convergence criterion 0.500D-04

Maximum number of steepest descent iterations 20

Maximum number of iterations for H1 2000

Convergence criterion for H1 0.100D-03

Input data file(s)

4.4Tw2noDssAllData.dat

Input data format FREE

SUMMARY OF DATA

Number of missing data patterns 123

COVARIANCE COVERAGE OF DATA

Minimum covariance coverage value 0.100

PROPORTION OF DATA PRESENT

Covariance Coverage

X1 X2 X3 X4 Y1

________ ________ ________ ________ ________

X1 0.539

X2 0.442 0.569

X3 0.322 0.423 0.536

X4 0.202 0.300 0.383 0.469

Y1 0.506 0.450 0.338 0.227 0.652

Y2 0.447 0.525 0.429 0.317 0.570

Y3 0.348 0.436 0.490 0.390 0.449

Y4 0.232 0.327 0.391 0.423 0.310

SEX 0.539 0.569 0.536 0.469 0.652

SES 0.539 0.569 0.536 0.469 0.652

Covariance Coverage

Y2 Y3 Y4 SEX SES

________ ________ ________ ________ ________

Y2 0.718

Y3 0.580 0.695

Y4 0.437 0.535 0.605

SEX 0.718 0.695 0.605 1.000

SES 0.718 0.695 0.605 1.000 1.000

SAMPLE STATISTICS

ESTIMATED SAMPLE STATISTICS

Means

X1 X2 X3 X4 Y1

________ ________ ________ ________ ________

4.974 4.996 4.610 4.231 4.511

Means

Y2 Y3 Y4 SEX SES

________ ________ ________ ________ ________

5.299 5.787 6.192 1.518 0.019

Covariances

X1 X2 X3 X4 Y1

________ ________ ________ ________ ________

X1 2.861

X2 1.978 3.299

X3 1.644 2.471 4.002

X4 1.404 1.894 2.997 4.491

Y1 0.518 0.671 0.664 0.593 0.765

Y2 0.468 0.651 0.685 0.612 0.530

Y3 0.402 0.529 0.651 0.598 0.439

Y4 0.388 0.508 0.639 0.584 0.415

SEX 0.072 0.079 0.098 0.189 0.018

SES 0.273 0.424 0.465 0.451 0.297

Covariances

Y2 Y3 Y4 SEX SES

________ ________ ________ ________ ________

Y2 0.639

Y3 0.409 0.474

Y4 0.401 0.367 0.455

SEX 0.023 0.008 0.010 0.250

SES 0.287 0.249 0.270 -0.006 1.107

Correlations

X1 X2 X3 X4 Y1

________ ________ ________ ________ ________

X1 1.000

X2 0.644 1.000

X3 0.486 0.680 1.000

X4 0.392 0.492 0.707 1.000

Y1 0.350 0.422 0.380 0.320 1.000

Y2 0.346 0.448 0.429 0.362 0.759

Y3 0.345 0.423 0.473 0.410 0.729

Y4 0.340 0.414 0.473 0.409 0.702

SEX 0.085 0.087 0.098 0.178 0.040

SES 0.153 0.222 0.221 0.202 0.323

Correlations

Y2 Y3 Y4 SEX SES

________ ________ ________ ________ ________

Y2 1.000

Y3 0.742 1.000

Y4 0.743 0.790 1.000

SEX 0.058 0.024 0.030 1.000

SES 0.342 0.344 0.380 -0.011 1.000

MAXIMUM LOG-LIKELIHOOD VALUE FOR THE UNRESTRICTED (H1) MODEL IS -22010.280

UNIVARIATE SAMPLE STATISTICS

UNIVARIATE HIGHER-ORDER MOMENT DESCRIPTIVE STATISTICS

Variable/ Mean/ Skewness/ Minimum/ % with Percentiles

Sample Size Variance Kurtosis Maximum Min/Max 20%/60% 40%/80% Median

X1 5.062 -0.900 1.000 6.54% 4.000 5.000 5.000

1452.000 2.810 0.175 7.000 21.49% 6.000 7.000

X2 5.021 -0.838 1.000 7.96% 4.000 5.000 5.000

1533.000 3.319 -0.242 7.000 25.24% 6.000 7.000

X3 4.620 -0.550 1.000 13.02% 2.000 5.000 5.000

1444.000 4.028 -0.926 7.000 20.84% 6.000 7.000

X4 4.256 -0.292 1.000 17.63% 2.000 4.000 5.000

1265.000 4.445 -1.268 7.000 17.79% 5.000 6.000

Y1 4.516 0.026 1.810 0.06% 3.770 4.310 4.530

1757.000 0.754 -0.105 7.593 0.06% 4.740 5.240

Y2 5.277 0.112 2.620 0.10% 4.640 5.090 5.278

1935.000 0.630 0.255 8.044 0.05% 5.469 5.929

Y3 5.759 0.094 3.220 0.05% 5.170 5.589 5.766

1872.000 0.462 0.004 8.212 0.05% 5.928 6.310

Y4 6.176 -0.006 3.555 0.06% 5.614 6.000 6.191

1631.000 0.452 0.436 8.906 0.06% 6.347 6.730

SEX 1.518 -0.063 1.000 48.24% 1.000 1.000 2.000

2695.000 0.250 -1.972 3.000 0.04% 2.000 2.000

SES 0.019 0.164 -2.823 0.04% -0.886 -0.358 -0.055

2695.000 1.107 -0.427 5.000 0.04% 0.250 0.989

THE MODEL ESTIMATION TERMINATED NORMALLY

MODEL FIT INFORMATION

Number of Free Parameters 52

Loglikelihood

H0 Value -16096.359

H0 Scaling Correction Factor 1.1218

for MLR

H1 Value -16090.644

H1 Scaling Correction Factor 1.1095

for MLR

Information Criteria

Akaike (AIC) 32296.717

Bayesian (BIC) 32603.473

Sample-Size Adjusted BIC 32438.253

(n* = (n + 2) / 24)

Chi-Square Test of Model Fit

Value 11.100*

Degrees of Freedom 8

P-Value 0.1961

Scaling Correction Factor 1.0297

for MLR

* The chi-square value for MLM, MLMV, MLR, ULSMV, WLSM and WLSMV cannot be used

for chi-square difference testing in the regular way. MLM, MLR and WLSM

chi-square difference testing is described on the Mplus website. MLMV, WLSMV,

and ULSMV difference testing is done using the DIFFTEST option.

RMSEA (Root Mean Square Error Of Approximation)

Estimate 0.012

90 Percent C.I. 0.000 0.027

Probability RMSEA <= .05 1.000

CFI/TLI

CFI 1.000

TLI 0.997

Chi-Square Test of Model Fit for the Baseline Model

Value 6422.860

Degrees of Freedom 44

P-Value 0.0000

SRMR (Standardized Root Mean Square Residual)

Value 0.014

MODEL RESULTS

Two-Tailed

Estimate S.E. Est./S.E. P-Value

IX BY

X1 1.000 0.000 999.000 999.000

X2 1.000 0.000 999.000 999.000

X3 1.000 0.000 999.000 999.000

X4 1.000 0.000 999.000 999.000

IY BY

Y1 1.000 0.000 999.000 999.000

Y2 1.000 0.000 999.000 999.000

Y3 1.000 0.000 999.000 999.000

Y4 1.000 0.000 999.000 999.000

WX1 BY

X1 1.000 0.000 999.000 999.000

WX2 BY

X2 1.000 0.000 999.000 999.000

WX3 BY

X3 1.000 0.000 999.000 999.000

WX4 BY

X4 1.000 0.000 999.000 999.000

WY1 BY

Y1 1.000 0.000 999.000 999.000

WY2 BY

Y2 1.000 0.000 999.000 999.000

WY3 BY

Y3 1.000 0.000 999.000 999.000

WY4 BY

Y4 1.000 0.000 999.000 999.000

WX2 ON

WX1 0.519 0.060 8.654 0.000

WY1 0.540 0.091 5.934 0.000

WX3 ON

WX2 0.605 0.049 12.337 0.000

WY2 0.439 0.131 3.356 0.001

WX4 ON

WX3 0.640 0.042 15.358 0.000

WY3 0.301 0.259 1.161 0.246

WY2 ON

WY1 0.309 0.031 10.015 0.000

WX1 0.032 0.012 2.625 0.009

WY3 ON

WY2 0.046 0.036 1.273 0.203

WX2 0.050 0.011 4.545 0.000

WY4 ON

WY3 -0.206 0.148 -1.395 0.163

WX3 0.085 0.018 4.854 0.000

X1 ON

SEX 0.293 0.082 3.562 0.000

SES 0.247 0.039 6.382 0.000

X2 ON

SEX 0.323 0.083 3.887 0.000

SES 0.384 0.040 9.680 0.000

X3 ON

SEX 0.399 0.093 4.310 0.000

SES 0.421 0.044 9.638 0.000

X4 ON

SEX 0.762 0.104 7.288 0.000

SES 0.411 0.048 8.556 0.000

Y1 ON

SEX 0.078 0.036 2.156 0.031

SES 0.269 0.018 15.042 0.000

Y2 ON

SEX 0.099 0.032 3.116 0.002

SES 0.260 0.016 16.686 0.000

Y3 ON

SEX 0.039 0.028 1.364 0.173

SES 0.225 0.015 15.175 0.000

Y4 ON

SEX 0.047 0.027 1.733 0.083

SES 0.244 0.013 18.374 0.000

IX WITH

IY 0.280 0.036 7.782 0.000

WX1 WITH

WY1 0.167 0.038 4.417 0.000

WX2 WITH

WY2 0.121 0.020 5.990 0.000

WX3 WITH

WY3 0.142 0.023 6.167 0.000

WX4 WITH

WY4 0.024 0.024 0.998 0.318

IY WITH

WY1 0.035 0.013 2.809 0.005

Means

IX 0.000 0.000 999.000 999.000

IY 0.000 0.000 999.000 999.000

WX1 0.000 0.000 999.000 999.000

WY1 0.000 0.000 999.000 999.000

Intercepts

X1 4.524 0.135 33.556 0.000

X2 4.499 0.136 33.074 0.000

X3 3.994 0.150 26.667 0.000

X4 3.067 0.170 18.006 0.000

Y1 4.386 0.058 75.986 0.000

Y2 5.144 0.051 100.939 0.000

Y3 5.723 0.045 126.348 0.000

Y4 6.116 0.044 137.926 0.000

WX2 0.000 0.000 999.000 999.000

WX3 0.000 0.000 999.000 999.000

WX4 0.000 0.000 999.000 999.000

WY2 0.000 0.000 999.000 999.000

WY3 0.000 0.000 999.000 999.000

WY4 0.000 0.000 999.000 999.000

Variances

IX 0.678 0.242 2.797 0.005

IY 0.303 0.015 19.676 0.000

WX1 2.102 0.262 8.031 0.000

WY1 0.308 0.019 16.168 0.000

Residual Variances

X1 0.000 0.000 999.000 999.000

X2 0.000 0.000 999.000 999.000

X3 0.000 0.000 999.000 999.000

X4 0.000 0.000 999.000 999.000

Y1 0.000 0.000 999.000 999.000

Y2 0.000 0.000 999.000 999.000

Y3 0.000 0.000 999.000 999.000

Y4 0.000 0.000 999.000 999.000

WX2 1.696 0.098 17.270 0.000

WX3 2.004 0.109 18.353 0.000

WX4 2.108 0.109 19.321 0.000

WY2 0.201 0.010 20.027 0.000

WY3 0.095 0.013 7.107 0.000

WY4 0.071 0.015 4.753 0.000

QUALITY OF NUMERICAL RESULTS

Condition Number for the Information Matrix 0.551E-04

(ratio of smallest to largest eigenvalue)

STANDARDIZED MODEL RESULTS

STDYX Standardization

Two-Tailed

Estimate S.E. Est./S.E. P-Value

IX BY

X1 0.486 0.087 5.571 0.000

X2 0.452 0.081 5.611 0.000

X3 0.413 0.074 5.539 0.000

X4 0.389 0.070 5.583 0.000

IY BY

Y1 0.630 0.017 36.280 0.000

Y2 0.690 0.015 46.798 0.000

Y3 0.807 0.020 40.344 0.000

Y4 0.811 0.018 46.190 0.000

WX1 BY

X1 0.856 0.050 17.288 0.000

WX2 BY

X2 0.859 0.042 20.323 0.000

WX3 BY

X3 0.878 0.036 24.514 0.000

WX4 BY

X4 0.880 0.032 27.925 0.000

WY1 BY

Y1 0.635 0.018 35.682 0.000

WY2 BY

Y2 0.608 0.014 44.162 0.000

WY3 BY

Y3 0.470 0.032 14.692 0.000

WY4 BY

Y4 0.440 0.028 15.771 0.000

WX2 ON

WX1 0.481 0.059 8.151 0.000

WY1 0.192 0.033 5.720 0.000

WX3 ON

WX2 0.540 0.046 11.700 0.000

WY2 0.122 0.037 3.297 0.001

WX4 ON

WX3 0.603 0.042 14.477 0.000

WY3 0.052 0.045 1.163 0.245

WY2 ON

WY1 0.353 0.034 10.260 0.000

WX1 0.096 0.037 2.576 0.010

WY3 ON

WY2 0.070 0.052 1.336 0.181

WX2 0.244 0.055 4.417 0.000

WY4 ON

WY3 -0.222 0.157 -1.412 0.158

WX3 0.501 0.115 4.367 0.000

X1 ON

SEX 0.087 0.024 3.584 0.000

SES 0.154 0.024 6.421 0.000

X2 ON

SEX 0.089 0.023 3.906 0.000

SES 0.222 0.023 9.790 0.000

X3 ON

SEX 0.100 0.023 4.337 0.000

SES 0.222 0.023 9.741 0.000

X4 ON

SEX 0.180 0.024 7.383 0.000

SES 0.204 0.024 8.610 0.000

Y1 ON

SEX 0.044 0.021 2.160 0.031

SES 0.324 0.021 15.724 0.000

Y2 ON

SEX 0.062 0.020 3.145 0.002

SES 0.343 0.019 17.775 0.000

Y3 ON

SEX 0.028 0.021 1.365 0.172

SES 0.347 0.022 15.708 0.000

Y4 ON

SEX 0.035 0.020 1.738 0.082

SES 0.378 0.019 20.302 0.000

IX WITH

IY 0.617 0.087 7.126 0.000

WX1 WITH

WY1 0.208 0.040 5.257 0.000

WX2 WITH

WY2 0.207 0.032 6.412 0.000

WX3 WITH

WY3 0.325 0.052 6.231 0.000

WX4 WITH

WY4 0.062 0.063 0.985 0.325

IY WITH

WY1 0.116 0.044 2.610 0.009

Means

IX 0.000 0.000 999.000 999.000

IY 0.000 0.000 999.000 999.000

WX1 0.000 0.000 999.000 999.000

WY1 0.000 0.000 999.000 999.000

Intercepts

X1 2.671 0.106 25.166 0.000

X2 2.472 0.095 26.147 0.000

X3 2.002 0.086 23.187 0.000

X4 1.451 0.087 16.711 0.000

Y1 5.019 0.107 47.025 0.000

Y2 6.439 0.133 48.553 0.000

Y3 8.387 0.146 57.588 0.000

Y4 9.007 0.179 50.269 0.000

WX2 0.000 0.000 999.000 999.000

WX3 0.000 0.000 999.000 999.000

WX4 0.000 0.000 999.000 999.000

WY2 0.000 0.000 999.000 999.000

WY3 0.000 0.000 999.000 999.000

WY4 0.000 0.000 999.000 999.000

Variances

IX 1.000 0.000 999.000 999.000

IY 1.000 0.000 999.000 999.000

WX1 1.000 0.000 999.000 999.000

WY1 1.000 0.000 999.000 999.000

Residual Variances

X1 0.000 999.000 999.000 999.000

X2 0.000 999.000 999.000 999.000

X3 0.000 999.000 999.000 999.000

X4 0.000 999.000 999.000 999.000

Y1 0.000 999.000 999.000 999.000

Y2 0.000 999.000 999.000 999.000

Y3 0.000 999.000 999.000 999.000

Y4 0.000 999.000 999.000 999.000

WX2 0.693 0.057 12.109 0.000

WX3 0.653 0.049 13.278 0.000

WX4 0.608 0.046 13.111 0.000

WY2 0.852 0.028 30.927 0.000

WY3 0.925 0.030 31.136 0.000

WY4 0.792 0.088 8.962 0.000

R-SQUARE

Observed Two-Tailed

Variable Estimate S.E. Est./S.E. P-Value

X1 1.000 999.000 999.000 999.000

X2 1.000 999.000 999.000 999.000

X3 1.000 999.000 999.000 999.000

X4 1.000 999.000 999.000 999.000

Y1 1.000 999.000 999.000 999.000

Y2 1.000 999.000 999.000 999.000

Y3 1.000 999.000 999.000 999.000

Y4 1.000 999.000 999.000 999.000

Latent Two-Tailed

Variable Estimate S.E. Est./S.E. P-Value

WX2 0.307 0.057 5.358 0.000

WX3 0.347 0.049 7.063 0.000

WX4 0.392 0.046 8.435 0.000

WY2 0.148 0.028 5.357 0.000

WY3 0.075 0.030 2.515 0.012

WY4 0.208 0.088 2.357 0.018

RESIDUAL OUTPUT

ESTIMATED MODEL AND RESIDUALS (OBSERVED - ESTIMATED)

Model Estimated Means

X1 X2 X3 X4 Y1

________ ________ ________ ________ ________

4.974 4.996 4.608 4.231 4.509

Model Estimated Means

Y2 Y3 Y4 SEX SES

________ ________ ________ ________ ________

5.299 5.786 6.192 1.518 0.019

Residuals for Means

X1 X2 X3 X4 Y1

________ ________ ________ ________ ________

0.001 -0.001 0.002 -0.001 0.001

Residuals for Means

Y2 Y3 Y4 SEX SES

________ ________ ________ ________ ________

0.000 0.000 -0.001 0.000 0.000

Standardized Residuals (z-scores) for Means

X1 X2 X3 X4 Y1

________ ________ ________ ________ ________

999.000 -0.764 999.000 999.000 0.913

Standardized Residuals (z-scores) for Means

Y2 Y3 Y4 SEX SES

________ ________ ________ ________ ________

0.086 2.901 -0.828 0.000 0.000

Normalized Residuals for Means

X1 X2 X3 X4 Y1

________ ________ ________ ________ ________

0.018 -0.021 0.034 -0.013 0.075

Normalized Residuals for Means

Y2 Y3 Y4 SEX SES

________ ________ ________ ________ ________

0.003 0.013 -0.051 0.000 0.000

Model Estimated Covariances

X1 X2 X3 X4 Y1

________ ________ ________ ________ ________

X1 2.868

X2 1.988 3.313

X3 1.588 2.471 3.983

X4 1.355 1.965 2.977 4.470

Y1 0.526 0.672 0.626 0.558 0.764

Y2 0.477 0.654 0.673 0.591 0.529

Y3 0.408 0.530 0.637 0.579 0.425

Y4 0.402 0.514 0.628 0.570 0.427

SEX 0.072 0.079 0.097 0.188 0.018

SES 0.272 0.423 0.464 0.450 0.298

Model Estimated Covariances

Y2 Y3 Y4 SEX SES

________ ________ ________ ________ ________

Y2 0.638

Y3 0.404 0.466

Y4 0.403 0.366 0.461

SEX 0.023 0.008 0.010 0.250

SES 0.287 0.249 0.270 -0.006 1.107

Model Estimated Correlations

X1 X2 X3 X4 Y1

________ ________ ________ ________ ________

X1 1.000

X2 0.645 1.000

X3 0.470 0.680 1.000

X4 0.378 0.511 0.706 1.000

Y1 0.356 0.423 0.359 0.302 1.000

Y2 0.353 0.450 0.422 0.350 0.758

Y3 0.353 0.427 0.468 0.401 0.713

Y4 0.349 0.416 0.464 0.397 0.719

SEX 0.085 0.086 0.097 0.178 0.041

SES 0.153 0.221 0.221 0.202 0.324

Model Estimated Correlations

Y2 Y3 Y4 SEX SES

________ ________ ________ ________ ________

Y2 1.000

Y3 0.741 1.000

Y4 0.743 0.789 1.000

SEX 0.058 0.025 0.031 1.000

SES 0.342 0.346 0.377 -0.011 1.000

Residuals for Covariances

X1 X2 X3 X4 Y1

________ ________ ________ ________ ________

X1 -0.007

X2 -0.009 -0.014

X3 0.056 0.000 0.019

X4 0.050 -0.071 0.020 0.021

Y1 -0.008 -0.002 0.038 0.035 0.002

Y2 -0.009 -0.003 0.012 0.021 0.001

Y3 -0.006 -0.002 0.014 0.019 0.014

Y4 -0.014 -0.006 0.011 0.014 -0.012

SEX 0.000 0.000 0.000 0.000 0.000

SES 0.001 0.001 0.001 0.000 -0.001

Residuals for Covariances

Y2 Y3 Y4 SEX SES

________ ________ ________ ________ ________

Y2 0.000

Y3 0.004 0.009

Y4 -0.002 0.002 -0.006

SEX 0.000 0.000 0.000 0.000

SES 0.000 0.000 0.000 0.000 0.000

Residuals for Correlations

X1 X2 X3 X4 Y1

________ ________ ________ ________ ________

X1 0.000

X2 -0.001 0.000

X3 0.016 0.000 0.000

X4 0.013 -0.018 0.001 0.000

Y1 -0.005 -0.001 0.021 0.018 0.000

Y2 -0.006 -0.001 0.006 0.012 0.000

Y3 -0.008 -0.004 0.005 0.008 0.016

Y4 -0.010 -0.001 0.010 0.011 -0.017

SEX 0.000 0.000 0.000 0.000 0.000

SES 0.001 0.001 0.000 0.000 -0.001

Residuals for Correlations

Y2 Y3 Y4 SEX SES

________ ________ ________ ________ ________

Y2 0.000

Y3 0.001 0.000

Y4 0.000 0.001 0.000

SEX 0.000 0.000 0.000 0.000

SES 0.000 -0.003 0.002 0.000 0.000

Standardized Residuals (z-scores) for Covariances

X1 X2 X3 X4 Y1

________ ________ ________ ________ ________

X1 -0.605

X2 999.000 999.000

X3 1.053 999.000 999.000

X4 1.195 -1.172 999.000 1.945

Y1 -0.732 -0.227 1.180 0.792 0.755

Y2 -0.996 -0.268 1.483 0.698 999.000

Y3 -0.374 -0.136 1.262 1.203 2.381

Y4 -0.890 -0.319 1.151 1.047 -2.482

SEX 999.000 999.000 999.000 999.000 999.000

SES 999.000 999.000 999.000 999.000 999.000

Standardized Residuals (z-scores) for Covariances

Y2 Y3 Y4 SEX SES

________ ________ ________ ________ ________

Y2 0.187

Y3 1.944 1.561

Y4 -0.876 999.000 -4.609

SEX 999.000 999.000 -0.273 0.000

SES 999.000 999.000 999.000 0.000 0.000

Normalized Residuals for Covariances

X1 X2 X3 X4 Y1

________ ________ ________ ________ ________

X1 -0.068

X2 -0.091 -0.127

X3 0.530 -0.001 0.181

X4 0.417 -0.625 0.178 0.194

Y1 -0.208 -0.039 0.819 0.640 0.064

Y2 -0.244 -0.076 0.306 0.472 0.058

Y3 -0.192 -0.052 0.419 0.509 0.886

Y4 -0.425 -0.170 0.308 0.392 -0.751

SEX -0.009 0.014 0.013 0.016 -0.013

SES 0.022 0.022 0.016 0.009 -0.042

Normalized Residuals for Covariances

Y2 Y3 Y4 SEX SES

________ ________ ________ ________ ________

Y2 0.022

Y3 0.316 0.565

Y4 -0.147 0.131 -0.335

SEX -0.006 -0.013 -0.020 0.000

SES -0.001 0.012 0.007 0.000 0.000

Beginning Time: 09:57:21

Ending Time: 09:57:22

Elapsed Time: 00:00:01

MUTHEN & MUTHEN

3463 Stoner Ave.

Los Angeles, CA 90066

Tel: (310) 391-9971

Fax: (310) 391-8971

Web: www.StatModel.com

Support: Support@StatModel.com

Copyright (c) 1998-2021 Muthen & Muthen
